# Increased ghrelin signaling prolongs survival in mouse models of human aging through activation of sirtuin1

**DOI:** 10.1038/mp.2015.220

**Published:** 2016-02-02

**Authors:** N Fujitsuka, A Asakawa, A Morinaga, M S Amitani, H Amitani, G Katsuura, Y Sawada, Y Sudo, Y Uezono, E Mochiki, I Sakata, T Sakai, K Hanazaki, T Yada, K Yakabi, E Sakuma, T Ueki, A Niijima, K Nakagawa, N Okubo, H Takeda, M Asaka, A Inui

**Affiliations:** 1 Department of Psychosomatic Internal Medicine, Kagoshima University Graduate School of Medical and Dental Sciences, Kagoshima, Japan; 2 Tsumura Research Laboratories, Tsumura, Ibaraki, Japan; 3 Division of Cancer Pathophysiology, National Cancer Center Research Institute, Tokyo, Japan; 4 Department of Digestive Tract and General Surgery, Saitama Medical Center, Saitama Medical University, Saitama, Japan; 5 Division of Life Science, Graduate School of Science and Engineering, Saitama University, Saitama, Japan; 6 Department of Surgery, Kochi Medical School, Kochi, Japan; 7 Department of Physiology, Jichi Medical University School of Medicine, Tochigi, Japan; 8 Department of Gastroenterology and Hepatology, Saitama Medical Center, Saitama Medical University, Saitama, Japan; 9 Department of Integrative Anatomy, Nagoya City University Graduate School of Medical Sciences, Nagoya, Japan; 10 Department of Physiology, Niigata University School of Medicine, Niigata, Japan; 11 Pathophysiology and Therapeutics, Faculty of Pharmaceutical Sciences, Hokkaido University, Sapporo, Japan; 12 Hokkaido University Hospital Gastroenterological Medicine, Sapporo, Japan; 13 Cancer Preventive Medicine, Hokkaido University Graduate School of Medicine, Sapporo, Japan

## Abstract

Caloric restriction (CR) is known to retard aging and delay functional decline as well as the onset of diseases in most organisms. Ghrelin is secreted from the stomach in response to CR and regulates energy metabolism. We hypothesized that in CR ghrelin has a role in protecting aging-related diseases. We examined the physiological mechanisms underlying the ghrelin system during the aging process in three mouse strains with different genetic and biochemical backgrounds as animal models of accelerated or normal human aging. The elevated plasma ghrelin concentration was observed in both klotho-deficient and senescence-accelerated mouse prone/8 (SAMP8) mice. Ghrelin treatment failed to stimulate appetite and prolong survival in klotho-deficient mice, suggesting the existence of ghrelin resistance in the process of aging. However, ghrelin antagonist hastened death and ghrelin signaling potentiators rikkunshito and atractylodin ameliorated several age-related diseases with decreased microglial activation in the brain and prolonged survival in klotho-deficient, SAMP8 and aged ICR mice. *In vitro* experiments, the elevated sirtuin1 (SIRT1) activity and protein expression through the cAMP–CREB pathway was observed after ghrelin and ghrelin potentiator treatment in ghrelin receptor 1a-expressing cells and human umbilical vein endothelial cells. Furthermore, rikkunshito increased hypothalamic SIRT1 activity and SIRT1 protein expression of the heart in the all three mouse models of aging. Pericarditis, myocardial calcification and atrophy of myocardial and muscle fiber were improved by treatment with rikkunshito. Ghrelin signaling may represent one of the mechanisms activated by CR, and potentiating ghrelin signaling may be useful to extend health and lifespan.

## Introduction

The increased lifespan of world population illustrates the success of modern medicine; however, the risk of developing many diseases increases exponentially with old age.^1^ Pharmacological therapies that delay or prevent aging-related diseases, such as dementia, cancer, diabetes mellitus, osteoporosis and vascular disease, are highly desired. Caloric restriction (CR) is known to retard aging and delay functional decline as well as the onset of diseases in most organisms.^2^ The beneficial effects of CR are considered to be mediated by several signaling pathways, including sirtuin (SIRT), insulin-like growth factor-1 (IGF-1)/insulin, adenosine monophosphate-activated protein kinase (AMPK) and mammalian target of rapamycin. Hypothalamic SIRT1 is required for normal response to CR^3^ and regulates energy balance, which is implicated in aging/longevity.^4, 5^ However, key regulated hormone activated by CR or precise mechanisms involved remain uncertain.

Ghrelin is an acylated hormone that is secreted mainly from stomach in response to fasting and CR.^6^ Ghrelin receptors (growth hormone secretagogue receptor type 1a; GHS-R1a) are expressed in several organs, including the brain and heart.^7^ Ghrelin has much broader physiological functions as an orexigen, including stimulation of growth hormone (GH) secretion,^8^ gastrointestinal motility^9^ and cardiac contraction,^10^ inhibition of energy metabolism^6^ and insulin secretion^11, 12^ and inhibition of inflammation^13^ and apoptosis. Orexigenic effect of ghrelin is mediated by activated AMPK in neuropeptide Y neurons in the hypothalamic arcuate nucleus.^14, 15, 16^ AMPK enhances SIRT1 activity by increasing cellular nicotinamide adenine dinucleotide (NAD^+^) levels and regulates energy expenditure.^17^ Therefore, we hypothesized that in CR ghrelin has a role in protecting aging-related diseases through a mediation of SIRT1.

The traditional Japanese Kampo medicine rikkunshito is able to potentiate ghrelin signaling by stimulating ghrelin secretion via serotonin 2b/2c receptor antagonism and by enhancing GHS-R activity.^18, 19, 20, 21, 22^ Of the 43 major chemical compounds contained in rikkunshito, atractylodin shows a marked increase in ghrelin/GHS-R-binding activity and ghrelin-induced cytosolic calcium ion (Ca^2+^) concentration in GHS-R-expressing cells through an allosteric mechanism. Atractylodin is a major ingredient detected in the plasma of rikkunshito-treated healthy volunteers.^23^

In this study, we examined the impact of ghrelin signaling on the survival of three mouse strains with different genetic and biochemical backgrounds. These animal models mimic accelerated or normal human aging. These models are useful in studying the common protective mechanisms regulating many age-related diseases. We show here that the elevated plasma acyl ghrelin concentration was observed in homozygous klotho mutant (kl/kl; klotho-deficient) and senescence-accelerated mouse prone/8 (SAMP8) mice. The treatment with ghrelin antagonist (D-Lys3)-GHRP-6 hastened death, whereas the ghrelin signaling potentiators rikkunshito and atractylodin, but not ghrelin, prolonged survival in klotho-deficient mice. Rikkunshito extended the survival of another pathological model of both SAMP8 and aged ICR mice, a model of normal aging. These results suggest the importance of potentiating endogenous ghrelin signaling or attenuation of ghrelin resistance in the extension of lifespan in animal models of human aging. Several age-related diseases were also ameliorated in all rodent models, including improved cardiac involvement in both klotho-deficient and SAMP8 mice and improved learning in aged ICR mice. The effects on survival were mostly independent of the orexigenic activity and interactions with insulin and IGF-1 signaling. As a protective mechanism against the aging process, ghrelin signaling was considered to be associated with increased SIRT1 activity and decreased microglial activation in the brain.

## Materials and methods

Klotho-deficient, SAMP8 and ICR mice as models of aging and GHS-R knockout mice were used. All experimental procedures were performed according to the ‘Guidelines for the Care and Use of Laboratory Animals' approved by each Laboratory Animal Committee. The detailed description of the materials and methods is provided in Supplementary Information.

## Results

### Role of ghrelin in aging process of klotho-deficient mice

A defect in klotho gene expression in mice leads to systemic age-dependent degeneration and a reduced lifespan. Multiple degenerations occur after 4 weeks of age, and premature death occurs at approximately 2 months of age.^24^ We examined the potential role of ghrelin signaling in klotho-deficient mice. We found that 5-week-old klotho-deficient mice showed an increase in acyl ghrelin, desacyl ghrelin, GH and corticosterone under fed or fasted conditions, while a decrease in acyl ghrelin/desacyl ghrelin (A/D) ratio suggestive of cachectic state, IGF-1, insulin and glucose were observed in klotho-deficient mice (Supplementary Figure S1a). The hypothalamic gene expression of neuropeptide Y and agouti-related peptide increased and proopiomelanocortin expression decreased in fasted klotho-deficient mice (Supplementary Figure S1b). These hormonal changes were consistent with those observed in cachexia.^18, 25^

**Figure 1:**
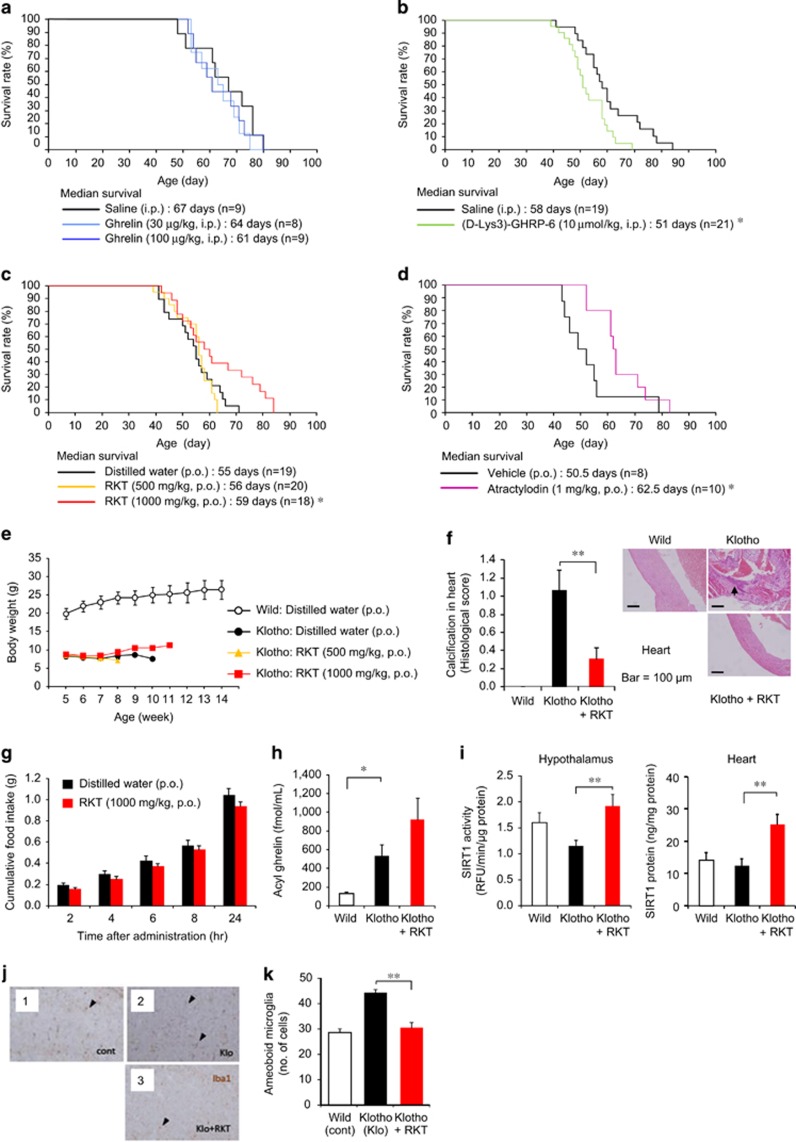
Role of ghrelin in aging in klotho-deficient mice. (**a** and **b**) Daily ghrelin (**a**) administration failed to prolong survival, but the growth hormone secretagogue receptor (GHS-R) antagonist (D-Lys3)-GHRP-6 (**b**) decreased median survival. *P<0.05. (**c–f**) Daily administration of rikkunshito (RKT) (**c**) and atractylodin (**d**) prolonged survival in klotho-deficient mice. (**e**) There was no effect of RKT on the weight loss of klotho-deficient mice. (**f**) Myocardial calcification, but not pericarditis, was observed in klotho-deficient mice. It was significantly decreased by RKT (1000 mg kg^−1^, p.o.) treatment. *P<0.05, **P<0.01. (**g**–**i**) There was no significant effect of RKT on food intake (**g**) for 24 h when klotho-deficient mice resided in individual houses. Four-day treatment with RKT (1000 mg kg^−1^, p.o.) did not affect plasma acyl ghrelin concentrations (**h**) but increased sirtuin1 (SIRT1) activity in the hypothalamus and SIRT1 protein expression in the heart (**i**) in klotho-deficient mice under the fed condition, which is used here because fasting is a severe stress leading to death in this model. *P<0.05, **P<0.01 (*n*=8–10). (**j** and **k**) The inflammatory activation of microglia in the brains of Klotho-deficient mice (Klo) was considerably reduced in the existence of RKT. (**k**) The number of aberrantly activated microglia with amoeboid morphology (arrow heads) decreased. **P<0.01 (*n*=14). cont; wild-type mice.

The plasma GH concentration, but not IGF-1, increased immediately after ghrelin administration (100 μg kg^−1^, intraperitoneally) in both klotho-deficient and age-matched wild-type mice (Supplementary Figure S2a). Klotho-deficient mice showed decreased food intake and body weight loss compared with wild-type mice, but ghrelin-induced increase in food intake or body weight was not observed in contrast to wild-type mice (Supplementary Figure S2b). These findings suggest that klotho-deficient mice have a marked resistance to the appetite-stimulating effect of ghrelin. Daily ghrelin administration (30 or 100 μg kg^−1^, intraperitoneally twice a day) failed to prolong survival, but ghrelin antagonist (D-Lys3)-GHRP-6 (10 μmol kg^−1^, intraperitoneally) decreased the median survival without changing body weight in klotho-deficient mice (Figures 1a and b and Supplementary Figure S2c). These findings indicate that endogenous ghrelin signaling is important in preventing premature death, but ghrelin resistance appears to cancel ghrelin's effect, similar to cancer cachexia.^18, 25^

Daily rikkunshito (1000 mg kg^−1^, p.o.) and atractylodin (1 mg kg^−1^, p.o.) administration significantly increased median survival (Figures 1c and d), without change in body weight or aging score in klotho-deficient mice (Figure 1e and Supplementary Figures S3a and b). A significant decrease in myocardial calcification was observed in rikkunshito-treated mice (Figure 1f and Supplementary Table S1). Rikkunshito (1000 mg kg^−1^, p.o.) did not affect 24-h food intake (Figure 1g) and failed to stimulate ghrelin secretion (Figure 1h) on day 4 in klotho-deficient mice. The other hormonal parameters, except insulin, were not changed by rikkunshito treatment (Supplementary Figure S3d). However, rikkunshito increased SIRT1 activity in the hypothalamus of klotho-deficient mice. The SIRT1 activity in the heart was difficult to determine owing to its extremely low levels, but a significant increase in heart SIRT1 protein by rikkunshito was observed in klotho-deficient mice (Figure 1i).

Klotho-deficient mice showed increased hypothalamic gene expression of interleukin-6 and tumor necrosis factor-α, which were not affected by rikkunshito and atractylodin administration (Supplementary Figure S4a). A microarray analysis of hypothalamic gene expression in klotho-deficient mice demonstrated that rikkunshito and atractylodin markedly improved the expression of age-related genes affecting inflammation, apoptosis, DNA repair and migration, with a modest change in proopiomelanocortin, IGF-1 and arginine vasopressin expression (Supplementary Figures S4b and c). Behavioral analyses could not be performed in klotho-deficient mice owing to their fragility.

### Antiaging effect of ghrelin signal potentiator rikkunshito in SAMP8 mice

**Figure 2:**
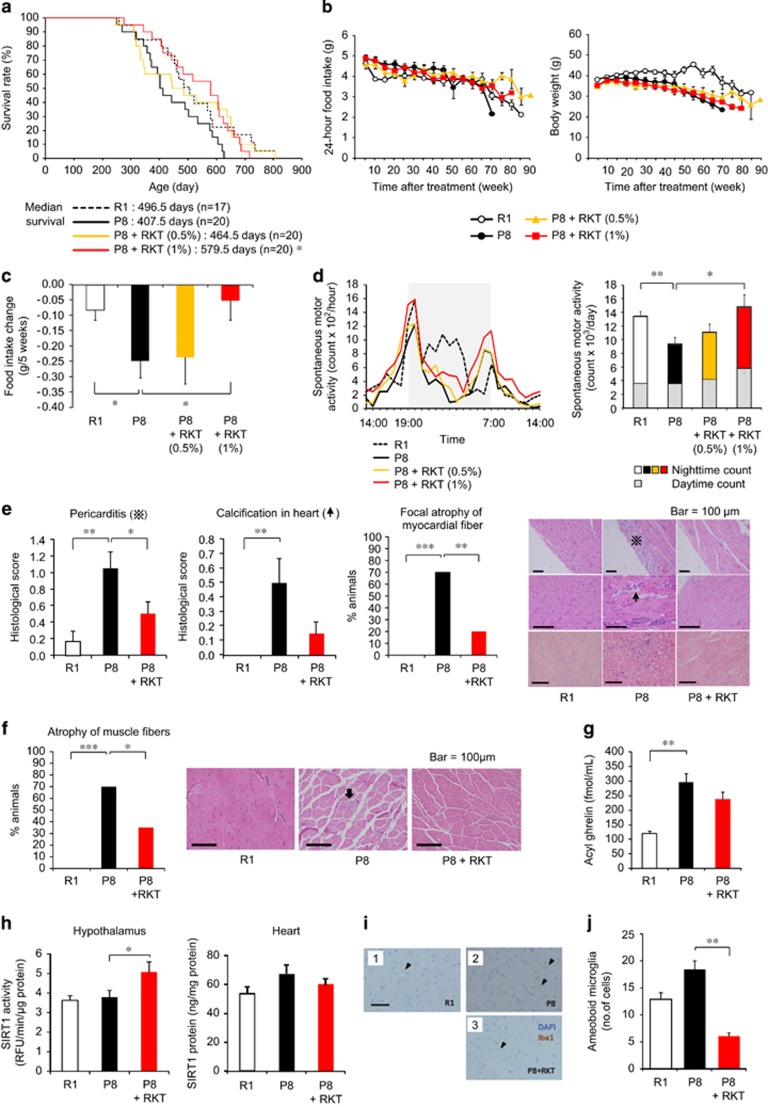
Antiaging effect of the ghrelin signaling potentiator rikkunshito in senescence-accelerated mouse prone/8 (SAMP8) mice. (**a**–**f**) Rikkunshito (RKT) improved the short lifespan (**a**) of SAMP8 (P8) mice. Decreases in food intake and body weight (**b**) were observed in both SAMP8 and SAMR1 (R1) mice during aging process, and RKT improved the rates of change in food intake (**c**) in SAMP8 mice. Thirty-nine-week-old SAMP8 mice exhibited decreased locomotor activity (**d**), which was recovered after rikkunshito administration. Pathological changes, such as pericarditis and atrophy of myocardial (**e**) and muscle fibers (**f**), at the end of the survival study were inhibited in RKT (1%)-treated mice. The mean dose of RKT was 600 mg kg^−1^ (0.5%) and 1240 mg kg^−1^ (1%) per day during the survival study. *P<0.05, **P<0.01 (*n*=17–20). (**g**) A 19-week treatment with RKT (1%) did not affect plasma acyl ghrelin concentrations in SAMP8 mice. **P<0.01 (*n*=15–19). (**h**) RKT (1000 mg kg^−1^, p.o. for 4 days) increased sirtuin1 (SIRT1) activity in the hypothalamus, but not SIRT1 protein expression in the heart, in 18-week-old SAMP8 mice. *P<0.05 (*n*=9–10). (**i** and **j**) RKT (1%) significantly ameliorated microglial pathological activation in the brains of SAMP8 (P8) mice, in which the number of Iba-1-positive amoeboid microglia (arrow heads) decreased. DAPI (4,6-diamidino-2-phenylindole) staining demonstrates the nucleus. The scale bar indicates 100 μm. **P<0.01 (*n*=14–16). R1; SAMR1 mice.

Senescence-accelerated mice have been inbred selectively based on age-associated pathological phenotypes. SAMP8 mice show age-related deficits and a shorter lifespan.^26^ The median survival of SAMP8 mice was shorter than senescence-accelerated mouse resistant/1 (SAMR1) mice; however, it was significantly increased by the daily rikkunshito (1%) administration (Figure 2a), with no change in the aging score (Supplementary Figure S5a). Decreases in food intake and body weight were observed in both SAMP8 and SAMR1 mice during aging process (Figure 2b). Food intake, body weight and food efficiency as expressed changes per 5 weeks were decreased in SAMP8 mice compared with SAMR1 mice. Rikkunshito improved food intake (Figure 2c and Supplementary Figure S5b). SAMP8 mice exhibited decreased locomotor activity, especially during the nighttime, which was recovered after rikkunshito administration (Figure 2d). There were no differences in anxiety-like behavior in an open-field test or memory disturbance in a step-through passive-avoidance test after rikkunshito treatment (Supplementary Figures S5c and d). Several pathological changes, such as pericarditis, atrophy of myocardial and muscle (sarcopenia) fiber (Figures 2e and f) and leukemia development (Supplementary Table S2), were significantly inhibited by the treatment.

Significant increases in the plasma concentrations of acyl ghrelin, desacyl ghrelin and GH were observed in 42-week-old SAMP8 mice compared with age-matched SAMR1 mice (Figure 2g and Supplementary Figure S6a). Increases in the hypothalamic gene expression of interleukin-1β, tumor necrosis factor-α and ionized calcium-binding adaptor molecule 1 (Iba-1), a microglia marker, were observed in SAMP8 mice (Supplementary Figure S6b). Rikkunshito treatment (1% for 19 weeks) increased plasma IGF-1 concentration but did not affect cytokines and Iba-1 expression (Supplementary Figure S6).

Gastric mucosal atrophy was observed in SAMP8 mice. The decrease in gastric pit thickness and the numbers of ghrelin-positive cells and the increase in activated macrophage were recovered by rikkunshito (Supplementary Figure S7). The treatment with rikkunshito increased SIRT 1 activity in the hypothalamus but had no effect on SIRT1 protein expression in the heart of SAMP8 mice (Figure 2h).

### Antiaging effect of rikkunshito in ICR and GHS-R knockout mice

**Figure 3:**
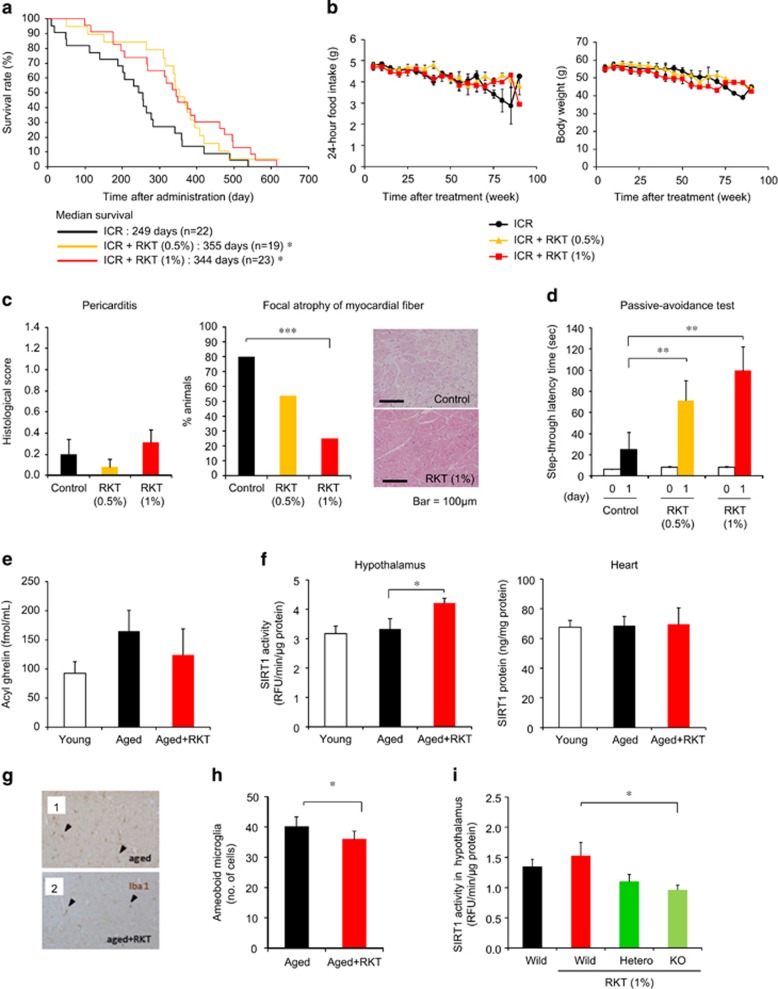
Antiaging effect of the ghrelin signaling potentiator rikkunshito in ICR and growth hormone secretagogue receptor (GHS-R) knockout mice. (**a**–**d**) Rikkunshito (RKT) prolonged the median survival (**a**) in ICR mice. Twenty-four-hour food intake and body weight (**b**) were not affected by RKT treatment. Pericarditis (**c**) was rarely observed, while focal atrophy of myocardial fiber was inhibited by RKT treatment in ICR mice after death. Two-month treatment with RKT facilitated the memory consolidation of passive avoidance learning (**d**) in ICR mice. The mean dose of RKT was 420 mg kg^−1^ (0.5%) and 850 mg kg^−1^ (1%) per day in this study. *P<0.05, **P<0.01 (*n*=19-23). (**e** and **f**). No significant change of plasma concentrations of acyl ghrelin (**e**) and SIRT1 (**f**) were observed between 4-month-old (young) ICR mice and 26-month-old (aged) ICR mice. Eight-month treatment with RKT (1%) did not affect plasma acyl ghrelin concentrations and sirtuin1 (SIRT1) protein expression in the heart but increased SIRT1 activity in the hypothalamus in aged ICR mice. *P<0.05 (*n*=6–8). (**g** and **h**) RKT significantly decreased the number of amoeboid microglia (arrow head) in the brains of aged ICR mice, while inflammatory activation of microglia maintained in the brains of control ICR mice. *P<0.05 (*n*=8). (**i**) Hypothalamic SIRT1 activity decreased in RKT-treated GHS-R knockout mice. *P<0.05 (*n*=5–7).

As a model of normal aging, 16- to 18-month-old ICR mice were used. They were assessed and grouped using aging scores and body weight reflecting growth curve because of a difference in the age of the animals available. Rikkunshito (0.5% and 1%) prolonged the median survival (Figure 3a), with no effect on food intake, body weight or aging scores in ICR mice (Figure 3b and Supplementary Figure S8a). This animal model exhibited the focal atrophy of myocardial fiber but not cardiac calcification and pericarditis, which was inhibited by rikkunshito (1%) treatment (Figure 3c and Supplementary Table S3). Rikkunshito facilitated the memory consolidation of passive avoidance learning in ICR mice 2 months after treatment (Figure 3d). There were no differences in anxiety-like behavior in the open-field and elevated plus-maze tests (Supplementary Figures S8b and c).

No significant change of plasma concentrations of acyl ghrelin was observed in 26-month-old aged ICR mice compared with 4-month-old young ICR mice. Eight-month treatment with rikkunshito did not affect plasma acyl ghrelin concentration and SIRT1 protein expression in the heart but increased SIRT 1 activity in the hypothalamus of aged ICR mice (Figures 3e and f).

Twelve-week-old GHS-R knockout, heterozygous and wild-type C57BL/6 mice were treated with rikkunshito (1%) containing chow or control chow for 4 weeks. Hypothalamic SIRT1 activity decreased in rikkunshito-treated GHS-R knockout mice (Figure 3i).

### Activation of SIRT1 pathway by ghrelin signaling

**Figure 4:**
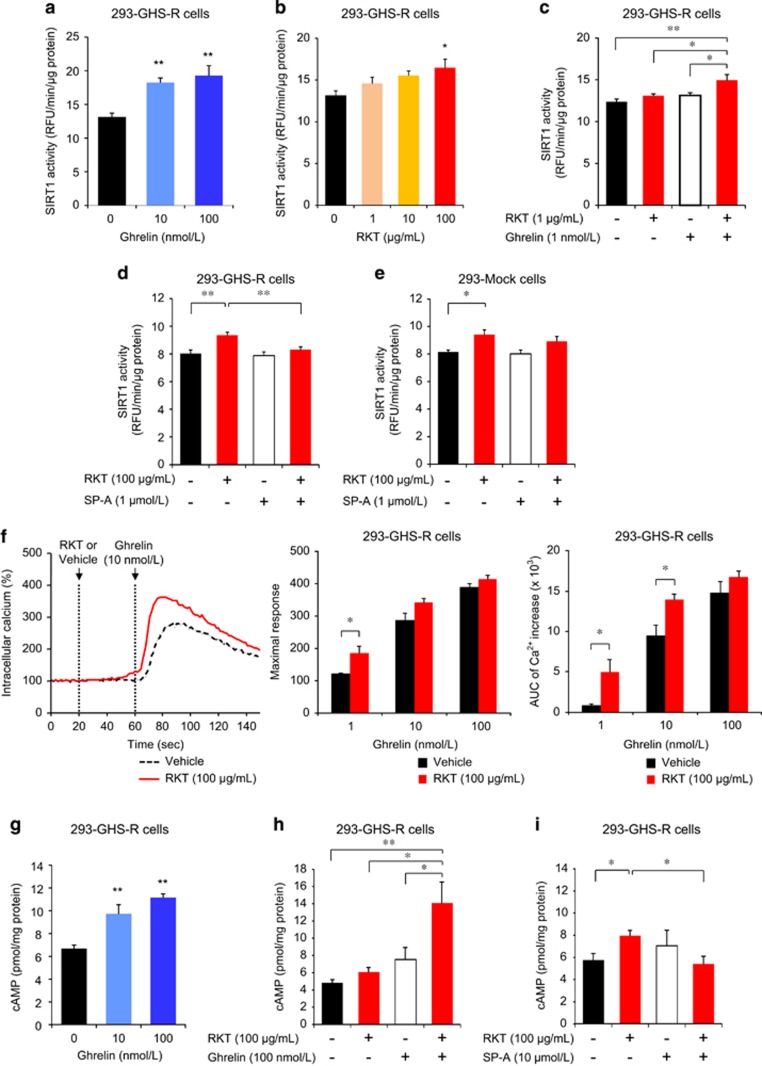
Activation of sirtuin1 (SIRT1) by ghrelin and rikkunshito (RKT) in growth hormone secretagogue receptor type 1a (GHS-R1a)-expressing HEK293 (293-GHS-R) cells. (**a**–**c**) Ghrelin (**a**) and RKT (**b**) increased SIRT1 activity in 293-GHS-R cells, which was enhanced by treatment with both ghrelin and RKT (**c**). *P<0.05, **P<0.01 (*n*=6). (**d** and **e**) Activation of SIRT1 by RKT was observed in 293-GHS-R cells (**d**) and mock cells (293-Mock) (**e**), which was inhibited by treatment with GHS-R inverse agonist (SP-A) in 293-GHS-R cells but not in mock cells. *P<0.05, **P<0.01 (*n*=6). (**f**) RKT potentiated ghrelin-induced intracellular Ca^2+^ flux in 293-GHS-R cells. *P<0.05 (*n*=4). (**g**–**i**) Ghrelin (**g**) increased cyclic adenosine monophosphate (cAMP) in 293-GHS-R cells, the effect of which was enhanced by treatment with RKT (**h**). RKT (**i**) increased cAMP in 293-GHS-R cells, which was inhibited by treatment with SP-A. *P<0.05, **P<0.01 (*n*=6).

We examined the cellular effects of ghrelin signaling on SIRT1 activity in GHS-R1a-expressing human embryonic kidney (HEK) 293 (293-GHS-R) cells. Ghrelin and rikkunshito increased SIRT1 activity in 293-GHS-R cells (Figures 4a and b), the effect of which was enhanced by treatment with both ghrelin and rikkunshito (Figure 4c).

GHS-R was found as a G-protein coupled receptor (GPCR) that initiates multiple intracellular signaling cascades.^27^ GPCRs coupled to Gαq/11 activate phospholipase C, which opens the endoplasmic inositol trisphosphate-gated Ca^2+^ channel and activates protein kinase C through production of diacylglycerol. On the other hand, GPCRs coupled to Gαs activate adenylyl cyclase, produce cyclic adenosine monophosphate (cAMP) and activate protein kinase A (PKA) and other downstream effectors. In this study, ghrelin elicited an increase in intracellular Ca^2+^ concentration in 293-GHS-R cells (Figure 4f). Rikkunshito had no effect on the intracellular Ca^2+^ in 293-GHS-R cells and mock-transfected (293-Mock) cells. However, it potentiated the ghrelin-induced Ca^2+^ flux in 293-GHS-R cells (Figure 4f), as we previously showed in COS cells and hypothalamic neurons^18, 19^ by rikkunshito and atractylodin. The impedance-based cell assay showed that rikkunshito potentiated the subsequent change in intracellular signaling induced by ghrelin, but not by vehicle, in 293-GHS-R cells (Supplementary Figure S9a). Gastric vagal afferent activity decreased in rats treated with ghrelin or rikkunshito. Similar effect was observed following the administration of its constituents atractylodes lancea rhizome, poria sclerotium and citrus unshiu peel (Supplementary Figure S10a).

**Figure 5:**
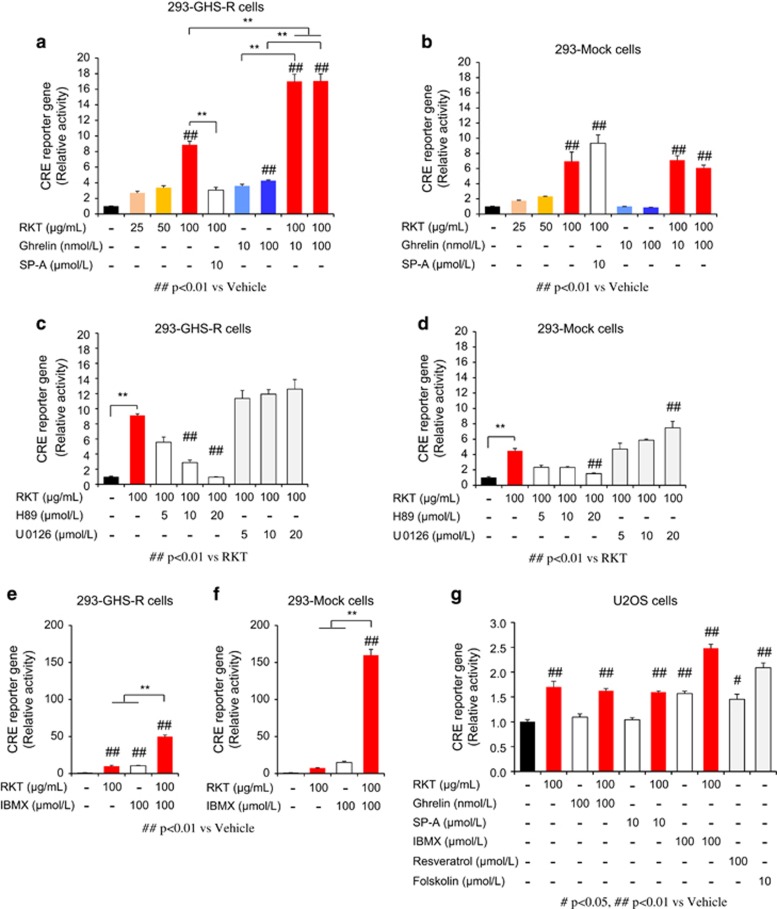
cAMP response element (CRE) reporter activity. (**a** and **b**) Rikkunshito (RKT) increased CRE reporter activity, which was enhanced by treatment with ghrelin and inhibited by treatment with growth hormone secretagogue receptor (GHS-R) inverse agonist (SP-A) in GHS-R1a-expressing HEK293 (293-GHS-R) cells (**a**) but not in mock cells (**b**). **P<0.01 (*n*=3). (**c**–**f**) RKT-induced CRE reporter activity was suppressed by protein kinase A inhibitor H89 but not by mitogen-activated protein kinase kinase inhibitor U0126 and enhanced by phosphodiesterase inhibitor 3-isobutyl-1-methylxanthine (IBMX) in 293-GHS-R cells (**c** and **e**) and mock cells (**d** and **f**). **P<0.01 (*n*=3). (**g**) In human osteosarcoma (U2OS) cells, RKT increased CRE reporter activity, which was not influenced by treatment with ghrelin or SP-A (*n*=3).

Ghrelin increased intracellular cAMP in 293-GHS-R cells, which was also enhanced by treatment with rikkunshito (Figures 4g and h). It was reported that cAMP response element binding (CREB) in the brain induced by CR upregulates SIRT1 gene transcription, resulting in neuroprotective responses that oppose brain aging.^28^ In the present study, ghrelin increased cAMP response element (CRE) reporter activity and phosphorylated CREB in 293-GHS-R cells and the effect was augmented by rikkunshito (Figure 5a and Supplementary Figure S11). These results suggest that ghrelin-induced SIRT1 activity is mediated by the cAMP–CREB pathway.

Rikkunshito increased cAMP (Figure 4i), CRE reporter activity (Figure 5a) and SIRT1 activity (Figure 4d) in 293-GHS-R cells without ghrelin treatment. These effects were inhibited by the treatment with GHS-R inverse agonist (D-Arg1, D-Phe5, D-Trp7,9, Leu11)-substance P (SP-A) (Figures 4d and i and 5a), suggesting the mediation by GHS-R that shows ligand-independent constitutive signaling.^29^ Rikkunshito-induced CRE reporter activity was suppressed by the PKA inhibitor H89 but not by the mitogen-activated protein kinase kinase inhibitor U0126 and enhanced by the phosphodiesterase inhibitor 3-isobutyl-1-methylxanthine (Figures 5c and e). Additionally, these effects of rikkunshito were observed in 293-Mock cells and human osteosarcoma (U2OS) cells (Figures 4e and 5b, d, f and g), suggesting a partial involvement of GHS-R-independent signaling related to cAMP/PKA pathway.

Next, the effect of ghrelin on SIRT1 was examined in human umbilical vein endothelial cells (HUVECs), which express both ghrelin and GHS-R1a.^30^ SIRT1 (ref. 31) and AMPK^32^ protect against age-associated vascular endothelial dysfunction. We found that rikkunshito elicited increases in SIRT1 activity and protein expression in HUVECs, as in ghrelin and atractylodin (Supplementary Figures S12a and b). These effects of rikkunshito and atractylodin were inhibited by treatment with (D-Lys3)-GHRP-6 or SP-A (Supplementary Figures S12a and c).

Calcium/calmodulin-dependent protein kinase kinase-β (CamKKβ) acts upstream of AMPK in mammalian cells.^33^ AMPK enhances SIRT1 activity by increasing cellular NAD^+^ levels, resulting in deacetylation and modulation of the activity of downstream SIRT1 targets.^17^ Activation of exchange protein directly activated by cAMP (Epac1), a cAMP effector protein, increases intracellular Ca^2+^ levels and activates the CamKKβ–AMPK pathway.^34^ Rikkunshito elevated phosphorylated AMPK levels, which was inhibited by the treatment with (D-Lys3)-GHRP-6 (Supplementary Figure S12d), suggesting the involvement of GHS-R signaling. Additionally, the SIRT1 protein expression levels in HUVECs were increased by the AMPK activator 5-amino-4-imidazolecarboxamide-1-beta-d-ribofuranoside and decreased by the AMPK inhibitor Compound C (Supplementary Figure S12e), suggesting that AMPK induces SIRT1 protein expression.

Ghrelin in combination with rikkunshito inhibited the hydrogen peroxide-induced apoptosis of 293-GHS-R cells, a model of the oxidative cell death (Supplementary Figure S9b).

### The inhibitory effect of SIRT1 pathway activation on microglia-mediated inflammation

Previous studies demonstrated that the activation of SIRT1 pathway ameliorated microglia-mediated inflammation through its inhibitory effect on receptor activator of nuclear factor-κB.^35^ To examine the possible involvement of SIRT1 in amelioration of microglial inflammation, rikkunshito was orally administered to klotho-deficient mice, an animal model of progeria, and microglial pathological activation in the dissected brains was morphologically analyzed. Rikkunshito significantly reduced the number of pathologically activated microglia with amoeboid morphology, which was predominantly observed in the brains of non-administered klotho-deficient mice (Figures 1j and k). In the brains of treated mice, the morphology of microglia was reversed to that of ramified resting microglia, which was featured with fine processes and few cytoplasm. Rikkunshito also significantly decreased the number of amoeboid microglia in the brains of both SAMP8 (Figures 2i and j) and aged ICR mice (Figures 3g and h).

## Discussion

We found that ghrelin antagonist (D-Lys3)-GHRP-6 hastened death, whereas the ghrelin signaling potentiators rikkunshito and atractylodin ameliorated several age-related diseases and prolonged survival in three mouse models of accelerated or normal human aging. These findings suggest that the elevated endogenous ghrelin signaling has an important role in preventing aging-related premature death.

Plasma acyl ghrelin concentrations were increased in both klotho-deficient and SAMP8 mice. It is most likely due to the anorexia and decreased body weight, because weight loss is a potent stimulus to ghrelin secretion in humans and animals.^36, 37^ Increased hypothalamic inflammatory cytokines observed in these mice may underlie the resistance to appetite-stimulating effects of ghrelin, the situation similar to cachexia associated with cancer and other diseases.^18, 25^ In SAMP8 mice only, rikkunshito improved in part the adaptive feeding and body weight response, suggesting that the effects on survival were mostly independent of the orexigenic activity.

Ghrelin, GHS and rikkunshito were reported to improve the GH–IGF-1 axis decline in aged animals and humans.^38, 39, 40, 41^ In this study of pathological aging, ghrelin increased GH but not IGF-1 in klotho-deficient mice and the GH–IGF-1 axis appeared not to downregulate in SAMP8 mice. Despite the studies indicating the importance of adequate levels of circulating GH and IGF-1 for healthy aging, the role of these anabolic hormones in the genesis of aging phenotypes and in the extension of lifespan remains highly controversial.^42^

Neurodegenerative disorders are one of the most potent risk factors for any age-related diseases. CR delays brain senescence and prevents neurodegeneration.^28, 43, 44^ In particular, hypothalamus has a programmatic role in the development of whole-body aging via immune-neuroendocrine integration.^45^ Enhanced ghrelin signaling improved the levels of neuronal genes involved in inflammation, apoptosis, DNA repair and migration in the hypothalamus of klotho-deficient mice. These findings are consistent with previous reports in which the beneficial effect of CR on age-related hypothalamic gene changes^46^ and the neuroprotective effect of ghrelin^47^ were observed. Cancer and dementia are the most debilitating conditions associated with aging. Rikkunshito facilitated memory consolidation of passive avoidance learning in aged ICR mice. The improved ghrelin signaling appears to protect against several age-associated neurodegenerative changes, including the pathology of brain and heart, as in CR.

Cardiovascular disease is one of the leading causes of death and disability in the aged society. Klotho gene polymorphisms in humans are associated with an altered risk for coronary artery disease.^48^ Klotho attenuates cellular apoptosis and senescence in vascular cells.^49^ CR attenuates oxidative stress and improves endothelial function in the aorta of aged F344 rats.^50^ Recently, ghrelin have been demonstrated to have beneficial effects in the cardiovascular system through a combination of direct and indirect actions.^10^ Ghrelin improves cardiac function and survival in heart failure using a mouse model of inherited dilated cardiomyopathy.^51^ Ghrelin inhibits sympathetic nervous system activity and stimulates GH secretion, which may help treat cardiovascular diseases and cardiac cachexia.^10, 52^ Histological analysis in this study revealed that pericarditis and calcification and focal atrophy of cardiac muscle developed in SAMP8 or aged ICR mice were improved by the treatment with rikkunshito. Calcification was also significantly attenuated by rikkunshito in klotho-deficient mice, suggesting a reason for the better prognosis. Ghrelin was reported to attenuate myocardial calcification induced by nicotine and vitamin D3 via an increase and decrease in osteopontin and endothelin-1 expression, respectively, in the myocardium.^53^ The mechanism through which leukemia, the main cause of death in SAMP8 mice, was inhibited by treatment with rikkunshito remains clarified in relation to ghrelin.

Changes in muscle mass during aging influence both lifespan and healthspan. The present study demonstrated improved atrophy of myocardial and muscle fiber by treatment with rikkunshito. These findings suggest ghrelin signaling improves age-related sarcopenia and could contribute to prolonged survival in mouse models. Rikkunshito administration recovered the decreased locomotor activity in SAMP8 mice. This result may also be mediated by improvement of muscle atrophy. Ghrelin and rikkunshito decreased the sympathetic nerve activity to brown adipose tissues. This finding suggests the reduced basal energy expenditure.

Ghrelin and rikkunshito increased SIRT1 activity and protein expression in 293-GHS-R cells and HUVECs through the GHS-R–cAMP–CREB pathway. GHS-Rs are expressed in several organs, including the hypothalamus and heart. Rikkunshito increased hypothalamic SIRT1 activity and SIRT1 protein expression in the heart in all three mouse models of aging. However, hypothalamic SIRT1 activity significantly decreased in rikkunshito-treated GHS-R knockout mice. These results suggest that SIRT1 activity in aged mice is mediated by ghrelin signaling.

Present findings that administration of rikkunshito to SAMP8, klotho-deficient and physiologically senescent mice resulted in the amelioration of microglial pathological activation are consistent with the previous report in which SIRT1 diminished the activation of nuclear factor-κB in microglia and the toxicity of amyloid β.^35^ Recent studies demonstrated that gut and intestine influenced the microglial behavior in the brain and determines the prognosis of some neurological disorders, such as multiple sclerosis; however, the pathophysiology has not been appreciated well.^45, 54^ Our findings indicate that ghrelin signaling activates SIRT1, and treatment for ghrelin resistance may exert a protective effect against brain and other organ/tissue pathologies during the process of aging through the SIRT1 pathway. These outcomes may be independent of the orexigenic activity of ghrelin^3, 55^ (Supplementary Figure S13).

CR has been established to extend the lifespan and improve the health in several species. A long-term CR study in rhesus monkeys demonstrated that CR lowered the incidence of aging-related deaths and delayed the onset of age-associated pathologies, such as diabetes, cancer, cardiovascular disease and brain atrophy,^56, 57^ although these results are still somewhat controversial. Many studies have shown that CR results in reduced oxidative stress, which is considered to be a key mechanism underlying the aging process.^58^ These CR effects are mediated by several signaling pathways, including SIRT, IGF-1/insulin, AMPK and mammalian target of rapamycin. These pathways may interact and may all have important roles in mediating different aspects of the response.^59^ Rapamycin has been shown to extend both the mean and maximum lifespan, and the development of newer, safer antiaging therapies based on this analog are expected.^60^

## Conclusion

The present study demonstrates that the key regulated hormones and mechanisms activated by CR should include ghrelin–GHS-R signaling pathways. The potentiation of ghrelin signaling may be an additional approach for the improvement of both healthspan and lifespan in modern aging societies.
