# Supplementary material for: Increased ghrelin signaling prolongs survival in mouse models of human aging through activation of sirtuin1
Source: Mol Psychiatry. 2016 Feb 2;21(11):1613–23. doi: 10.1038/mp.2015.220 (PMC5078860; doi:10.1038/mp.2015.220)
Supplement: Supplementary Information [file mp2015220x1.pdf]

Materials and Methods

Animals

Three mouse strains with different genetic and biochemical backgrounds (klotho-deficient mice, SAMP8 mice, and ICR mice) were used as animal models of aging. Four-week-old male klotho-deficient mice and age-matched wild-type mice were obtained from Japan Clea (Tokyo, Japan). Male SAMP8 and the reference to the control strain, SAMR1 mice, were obtained from Japan SL (Shizuoka, Japan). Twelve-week-old male mice and 16- toC 18-month-old male ICR mice weighing 33-68 g were obtained from Charles River Japan (Yokohama, Japan). Mice were housed in a regulated environment and given free access to water and standard laboratory chow. All experimental procedures were performed according to the "Guidelines for the Care and Use of Laboratory Animals" approved by each laboratory committee.

Reagent preparation

Ghrelin (acyl ghrelin, Peptide Institute, Osaka, Japan) and GHS-R antagonist (D-Lys3)-GHRP-6 (Bachem California, CA, USA) were dissolved in saline. The Kampo medicine rikkunshito (Tsumura, Tokyo, Japan) is a dried powder from herbal extract composed of the following eight constituents: Atractylodes lancea rhizome (*Atractylodis lanceae rhizoma*), Ginseng (*Ginseng radix*), Pinellia Tuber (*Pinelliae tuber*), Poria Sclerotium (*Poria*), Jujube (*Zizyphi fructus*), Citrus Unshiu Peel (*Aurantii nobilis pericarpium*), Glycyrrhiza (*Glycyrrhizae radix*), and Ginger (*Zingiberis rhizoma*). This extract was suspended in distilled water at doses of 500 and 1000 mg/kg for p.o. administration. Atractylodin (Tsumura), an active component of rikkunshito, was dissolved in a 0.1% ethanol and 1% Tween-80 solution.

Animal experiments (Klotho-deficient mice)

Experiment 1: Briefly, 5-week-old klotho-deficient and wild-type mice were divided into an overnight food-deprived group and free-fed group. Under ether anesthesia, blood samples were collected using a syringe containing aprotinin and ethylenediaminetetraacetic acid (EDTA)-2Na and centrifuged for 3 min at 10,000 rpm. For the ghrelin assay, 10% 1 mol/L

hydrochloric acid (HCl) was added to the plasma obtained. Tissue samples were collected and immediately frozen in liquid nitrogen. After processing, all sample aliquots were stored at -80ºC until measurement.

Experiment 2: Ghrelin (100 μg/kg) was intraperitoneally injected into klotho-deficient and wild-type mice, and blood samples were collected at 10, 30, and 60 min after administration.

Experiment 3: Ghrelin (100 μg/kg, i.p.) was injected into klotho-deficient and wild-type mice twice a day (morning and evening) for 4 days. On day 1, food intake in individual houses was measured at 1 and 24 hours after injection. Body weight was measured daily for 4 days.

Experiment 4: Ghrelin (30 and 100 μg/kg, i.p. twice a day), (D-Lys3)-GHRP-6 (10 μmol/kg, i.p.), rikkunshito (500 and 1000 mg/kg, p.o.), and atractylodin (1 mg/kg, p.o.) were administered daily to 5-week-old klotho-deficient mice until 100 days old. Body weight and food intake were measured. The median survival was calculated using a Kaplan-Meier plot. After death or euthanasia at the end of the survival study, tissue samples (heart and brain) were collected for the histochemical analysis.

Experiment 5: Rikkunshito (1000 mg/kg) was orally administered to klotho-deficient mice for 4 days. On day 1, food intake in individual houses was measured for 24 hours after administration. On day 4, blood and tissue samples for SIRT1 analysis were collected 2 hours after administration.

Experiment 6: Rikkunshito (1000 mg/kg) and atractylodin (1 mg/kg, p.o.) were orally administered to klotho-deficient mice for 11 days, and hypothalamic samples were collected for cytokine assay and a microarray analysis.

Animal experiments (SAMP8 mice)

Experiment 1: 23-week-old SAMP8 mice were given rikkunshito (0.5%, 1%)-containing chow or control chow in individual houses. Body weight and food intake were measured. 24-hour locomotor activity was monitored, and the open-field test and step-through passive-avoidance test were performed at 16 and 17 weeks after treatment. Median survival was calculated using Kaplan-Meier plots. After death in the survival study, heart samples were collected for the histochemical analysis.

Experiment 2: Fasting blood and tissue samples (stomach and brain) for

immunohistochemical and gene expression studies were collected at 19 weeks after treatment with rikkunshito in SAMP8 mice.

Experiment 3: Rikkunshito (1000 mg/kg, p.o.) was daily administered to 18-week-old SAMP8 mice for 4 days, and tissue samples were collected 2 hours after administration for SIRT1 analysis.

Animal experiments (ICR mice)

Experiment 1: In this experiment, 16- to 18-month-old ICR mice were given rikkunshito (0.5, 1%)-containing chow or control chow in individual houses. Because of a large difference in age, the aged group of ICR mice was retrospectively assessed using a grading score with accelerating aging (> 1.0) and body weight (> 50 g) at the start of the study. At 2 months after treatment with rikkunshito, a passive avoidance test, the elevated plus-maze test, and the open-field test were performed. Median survival was calculated using a Kaplan-Meier plot. After death in the survival study, heart samples were collected for the histochemical analysis.

Experiment 2: Fasting blood and tissue samples for SIRT1 analysis were collected in 26-month-old ICR mice treated with rikkunshito (1%) for 8 months or 4-month-old ICR mice.

Experiment 3: Brain samples for immunohistochemical study were collected 4 weeks after treatment with rikkunshito (1%) in 12-month-old ICR mice.

Animal experiments (GHS-R knockout mice)

Twelve-week-old GHS-R knockout mice, heterozygous mice, and wild type C57BL/6 mice were treated with rikkunshito (1%)-containing chow or control chow. After 4 weeks, hypothalamic samples in these mice were collected.

Analytical assays

The levels of acyl and desacyl ghrelin (Mitsubishi Chemical Medience, Tokyo, Japan), GH (Millipore Corporation, Billerica, MA, USA), insulin (Morinaga Institute of Biological Science Inc, Kanagawa, Japan), IGF-1 (Assaypro LLC, St. Charles, MO, USA), corticosterone (Assaypro LLC), glucose (Wako Pure Chemical Industries, Osaka, Japan), insulin-like growth factor-binding protein (IGFBP-3; R&D Systems, Minneapolis, MN,

USA), and leptin (BioVendor LLC, Candler, NC, USA) were measured using an enzyme-linked immunosorbent assay (ELISA) or a colorimetric assay. SIRT1 activity was measured using CycLex SIRT1/Sir2 Deacetylase Fluorometric Assay kit (CycLex Co., Ltd, Ina, Nagano, Japan). Level of mouse SIRT1 in tissue sample was measured using an ELISA (Cusabio, Hubei, China). Total protein was measured using BCA Protein Assay Reagent (Thermo Fisher Scientific K.K., Kanagawa, Japan).

Gene expression assay

Hypothalamic gene expression levels were measured using a microarray analysis (Agilent Expression Array, Takara Bio Inc. Shiga, Japan) and a real-time polymerase chain reaction system (ABI 7900HT, Applied Biosystems, CA, USA). Total ribonucleic acid (RNA) was extracted from the hypothalamic block using an RNeasy kit (Qiagen, CA, USA), and DNA was removed from total RNA using RNase-Free DNase (Qiagen). Reverse transcription reactions were performed using a TaqMan reverse transcription kit (Applied Biosystems). All oligonucleotide primers and fluorogenic probe sets for TaqMan real-time PCR were obtained from Applied Biosystems: NPY (Mm00445771_m1), AgRP (Mm00475829_g1), POMC (Mm00435874_m1), orexin A (Mm01964030_s1), corticotropin-releasing factor (Mm01293920_s1), and prepro-ghrelin (Mm00445450_m1), Interferon-γ (Mm01168134_m1), IL-1β (Mm01336189_m1), IL-6 (Mm00446190_m1), and TNF-α (Mm00443259_g1), Iba-1/Aif-1 (Mm00479862_g1), Peripheral-type benzodiazepine receptor (Tspo) (Mm00437828_m1), and Glyceraldehyde-3-phosphate dehydrogenase (GAPDH) (Mm99999915_g1) was used as an endogenous control.

Food intake, body weight, and aging score

The rate of change in food intake, body weight, food efficiency (calculated as body weight gain per food intake every five weeks), and grading score with accelerating aging during the survival study were obtained from least squares analysis.

Locomotor activity

The locomotor activity of mice in the home cage was measured during

a light–dark cycle with lights on from 7:00 to 19:00 with an infrared sensor (NS-AS01; Neuroscience, Inc., Tokyo, Japan).

Step-through passive-avoidance test

The apparatus (Neuroscience, Inc.) for the step-through passive-avoidance test consisted of two compartments, one illuminated (100 mm x 120 mm x 145 mm) and the other dark (140 mm x 185 mm x 190 mm), which were separated by a guillotine door. A mouse was placed in the illuminated compartment and stepped through the open guillotine door into the dark compartment and was given a foot shock (0.3 mA) for three seconds. Such trials were performed once a day, and the time spent in the illuminated compartment was defined as the latency period.

Open-field test

The open-field test consisted of a square floor (50 cm × 50 cm) enclosed by walls 25 cm high and divided into 25 areas of 10 cm intervals. A mouse was placed in the center part of the open field, and the total number of line crossings in areas and total number of entries into the central part for 5 min were determined using the analysis software LimeLight (Neuroscience, Inc.).

Elevated plus-maze test

The elevated plus-maze test consisted of two open arms and two enclosed arms (20 cm × 5 cm each), arranged so that the arms of the same type were opposite each other and elevated to a height of 50 cm. A mouse was placed in the central square of the elevated plus-maze, and the number of entries into the open arms and the time spent in the open arms in the plus-maze in 5 min were measured using the analysis software LimeLight.

Histochemical study

After death or euthanasia at the end of the survival study, several tissue samples including heart and gastrocnemius muscle were fixed in 10% phosphate-buffered formalin, paraffin embedded, and stained with hematoxylin and eosin for light microscopic examination. Scores were obtained using a semi-quantitative pathological scoring system (None; 0, Minimal; +1, Mild; +2, Moderate; +3).

For brain samples, serial sections of 10 μm thickness were mounted onto MAS-coated glass slides, dewaxed with xylene and processed through ethanols to water. The sections were subsequently incubated with anti-Iba-1 rabbit polyclonal antibody (Wako Pure Chemical Industries), and then processed according to the peroxidase-labeled antibody method. The products were visualized in a reaction with 3.3'-diamino-benzidine (DAB) and H_2O_2. Stained sections were observed equipped under a light microscope with a color-chilled 3CCD camera. The number of amoeboid microglia positively stained with anti-Iba1 antibody was quantitatively analyzed under a light microscope. The five 400 x 600 μm squares within the identical brain area (2 mm square) beneath the corpus callosum of mice were blindly captured, and the number of activated microglia with amoeboid morphology were counted and analyzed statistically (Prism 6, GraphPad Software Inc., La Jolla, CA).

Electrophysiologic study

The afferent activity of the gastric vagus nerve and the sympathetic nerve activity of the brown adipose tissues in urethane anesthetized rats were recorded via a pair of silver wire electrodes. A rate meter with a rest time of 5 s was used to observe the time course of nerve activity. Ghrelin (10 ng/rat) and rikkunshito (1000 mg/kg) or its constituents (400 mg/kg) were administered through a catheter inserted into the inferior vena cava and the duodenum, respectively. The mean numbers of impulses per 5 s over 50 s before and after the injection were compared.

Cell culture and transfection

293-GHS-R cells and 293-Mock cells, which had been stably transfected with the expression vector of C-terminal FLAG-tagged human GHS-R1a or empty vector, respectively, were cultured in Dulbecco's modified Eagle's medium (DMEM) (Wako Pure Chemical Industries) supplemented with 10% fetal bovine serum (FBS) (Invitrogen, Carlsbad, CA, USA) at 37ºC under 5% CO_2 in air. Transfection was performed by using PEI Max (Polysciences, Inc., Warrington, PA).

SIRT1 activity assay

293-GHS-R cells or 293-Mock cells were seeded in 24-well plates and

cultured for 24-hour. The media was changed to serum-free DMEM and incubated overnight. After that, the cells were pretreated with rikkunshito for 1 h, and then stimulated with ghrelin for an additional 6 h. SIRT1 activity in the cell lysates was measured using CycLex SIRT1/Sir2 Deacetylase Fluorometric Assay kit (CycLex Co., Ltd).

Ca^{2+} flux assay

A Ca^{2+} flux assay was performed using HEK293A cells that stably expressed human GHS-R1a and mock cells. The cells were seeded in 96-well plates and treated with ghrelin (1-100 nmol/L), rikkunshito (100 μg/mL), or vehicle. The intracellular Ca^{2+} was measured with the FLIPR Calcium 5 Assay kit (R8185, Molecular Devices, LLC, Sunnyvale, CA, USA) in accordance with the manufacturer's instructions. The increase in maximal response and the area under the curve (AUC) of Ca^{2+} were evaluated.

cAMP assay

293-GHS-R cells were seeded in 24-well plates and cultured for 24-hour. The media was changed to serum-free DMEM and incubated overnight. After that, the cells were pre-treated with 300μM IBMX for 30 min, followed by ghrelin for 30 min, and rikkunshito and SP-A for 90 min in the presence of IBMX. The intracellular cAMP concentrations were measured with a Direct cAMP ELISA kit (Enzo Life Sciences, Farmingdale, NY, USA) in accordance with the manufacturer's instructions.

Reporter gene assay

293-GHS-R or 293-Mock cells seeded in 24-well plate were transiently transfected with pGL4.29 [luc2P/CRE/hygro] (Promega, Madison, WI) (200 ng) and pGL4.75 [hRLuc-CMV] (Promega) (1 ng). Twenty-four hours after transfection, the media was changed to serum-free DMEM and incubated overnight. After that, the cells were pretreated with 100 μg/ml rikkunshito for 1 h, and then stimulated with 100 nM ghrelin (Peptide Institute Inc., Osaka, Japan) for an additional 6 h. Luciferase activities were measured using Dual-Luciferase Reporter Assay System (Promega) and an AB-2000 luminescencer-PSN (Atto, Tokyo, Japan).

Human umbilical vein endothelial cells (HUVECs)

HUVECs (Lonza, Walkersville, MD, USA) at 3-6 passages were used in experiments. The cells were plated (3 x 10^5 cells/well) into 6-multiwell plates and cultured for 24 hours in EGM-2 medium (Lonza) at 37°C in a humidified atmosphere of 95% air and 5% CO_2. After 24 hours of pre-incubation in serum-free culture medium, the cells were cultured for another 24 hours in the presence of vehicle (0.1% dimethylsulfoxide) or the test substances human acyl ghrelin, (D-Lys3)-GHRP-6, SP-A (Sigma-Aldrich, St. Louis, MO, USA), rikkunshito, atractylodin, 5-Amino-4-imidazolecarboxamide-1-beta-D-ribofuranoside (AICAR; Wako Pure Chemical Industries), and Compound C (Sigma-Aldrich).

After washing with phosphate-buffered saline, the cells were treated with Lysis Buffer (AdipoGen International, Inc., San Diego, CA, USA) for 5 min. After processing, the obtained cell lysate was stored at -80°C until measurement. Levels of SIRT1 protein (intracellular, human, AdipoGen International, Inc.) and phosphorylated AMPK (AMPKALPHA PT172, Invitrogen, Life Technologies, Grand Island, NY, USA) were measured using an ELISA.

Impedance-based cell assay

The impedance-based cell assay was performed using the CellKey™ system (Molecular Devices, LLC). The CellKey™ assay system can detect electrical impedance across monolayer cells embedded in electrical fields in each well of 96-well dishes, and these changes indicate changes in intracellular signaling. Ghrelin and rikkunshito were applied to 96-well plates seeded with human GHS-R1a-expressing HEK293A cells, and agonist-induced changes in cellular impedance were measured with the system.

Caspase-3/7 activity assay

GHS-R1a-expressing HEK293A and mock cells were exposed to H_2O_2 (0.15 mmol/L) for 20 hours. Cell apoptosis was determined with a caspase-3/7 activity assay using IncuCyte (Essen BioScience, Inc, Ann Arbor, MI, USA). The data are expressed as the ratio of fluorescence intensity in caspase-3/7-positive cells treated with 100 nmol/L ghrelin and/or 100 μg/mL rikkunshito.

Western blotting

293-GHS-R1a cells were pre-treated with 100 μg/mL rikkunshito for 60 min. After that, the cells were stimulated with 100 nmol/L ghrelin for an additional 30 min in the presence of IBMX and rikkunshito. The cells were washed with ice-cold Tris-buffered saline (TBS) and lysed with sodium dodecyl sulfate (SDS)-sample buffer (25 mM Tris-HCl (pH 6.8), 4% glycerol, 0.8 % SDS, 2% 2-mercaptoethanol, and 0.0002% bromophenol blue). For western blotting, the lysates were fractionated by SDS–polyacrylamide gel electrophoresis and transferred onto Immobilon-P membranes (Millipore). The membranes were blocked with 5% nonfat dry milk in TBS containing 0.05% Tween 20 (TBS-T) and incubated with the following antibodies: anti-CREB, and anti-phospho CREB (Cell Signaling, Danvers, MA, USA); anti-SIRT1 (Sigma); anti-actin (Millipore). After washing with TBS-T, the membranes were incubated with horseradish peroxidase-conjugated antibody against mouse or rabbit IgG (Jackson Immuno Research Laboratories, West Grove, PA, USA). The blots were detected by Immobilon Western HRP Substrate detection reagents (Millipore) using a LAS 4010 system (GE Healthcare Life Sciences, Buckinghamshire, UK).

Statistical analysis

Sample size was based on preliminary experiment. Animals were randomly allocated to experimental groups to be no difference in the body weight. Animal studies excluded aging score were not blinded. Values for individual groups are shown as the mean ± standard error (SE). To assess differences among groups, a Student *t*-test, a multi-group Dunnett test, a Bonferroni test, or Chai-square test for independence was performed. Mortality data were compared with log-rank tests and Gehan-Breslow-Wilcoxon tests. Values of $P < 0.05$ were considered statistically significant.

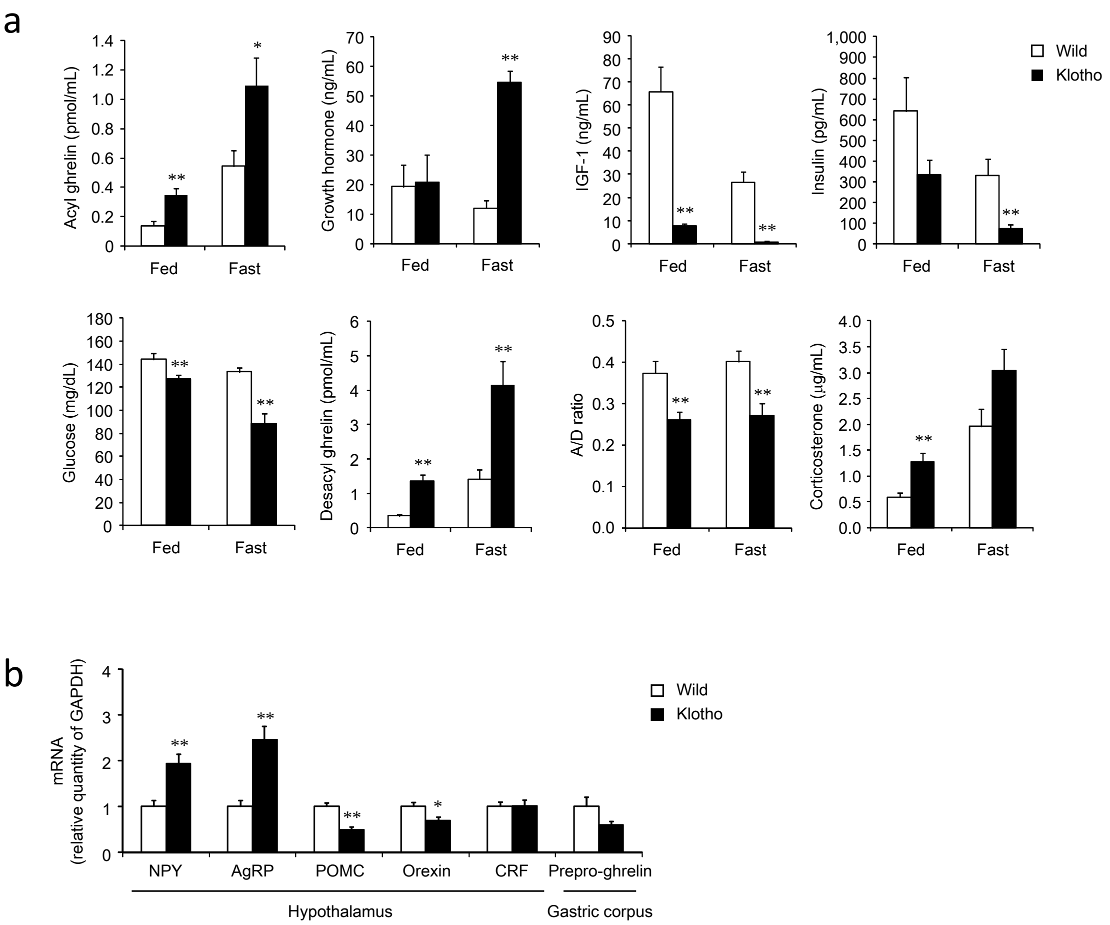

Supplementary Figure1. Ghrelin-related factors in klotho-deficient mice. (**a**) The plasma concentrations of acyl ghrelin, growth hormone (GH), desacyl ghrelin, and corticosterone increased, and the insulin-like growth factor (IGF-1), insulin, glucose, and acyl ghrelin/desacyl ghrelin (A/D) ratio decreased in 5-week-old klotho-deficient mice under fed or/and fasted conditions. These hormonal changes were consistent with those observed in cachexia. (**b**) The hypothalamic gene expression of neuropeptide Y (NPY) and agouti-related peptide (AgRP) increased, and proopiomelanocortin (POMC) and orexin expression decreased in fasted klotho-deficient mice, suggesting some response to starvation that failed to ameliorate the cachectic condition. CRF: corticotropin-releasing factor. * $P < 0.05$, ** $P < 0.01$ (n=9-11).

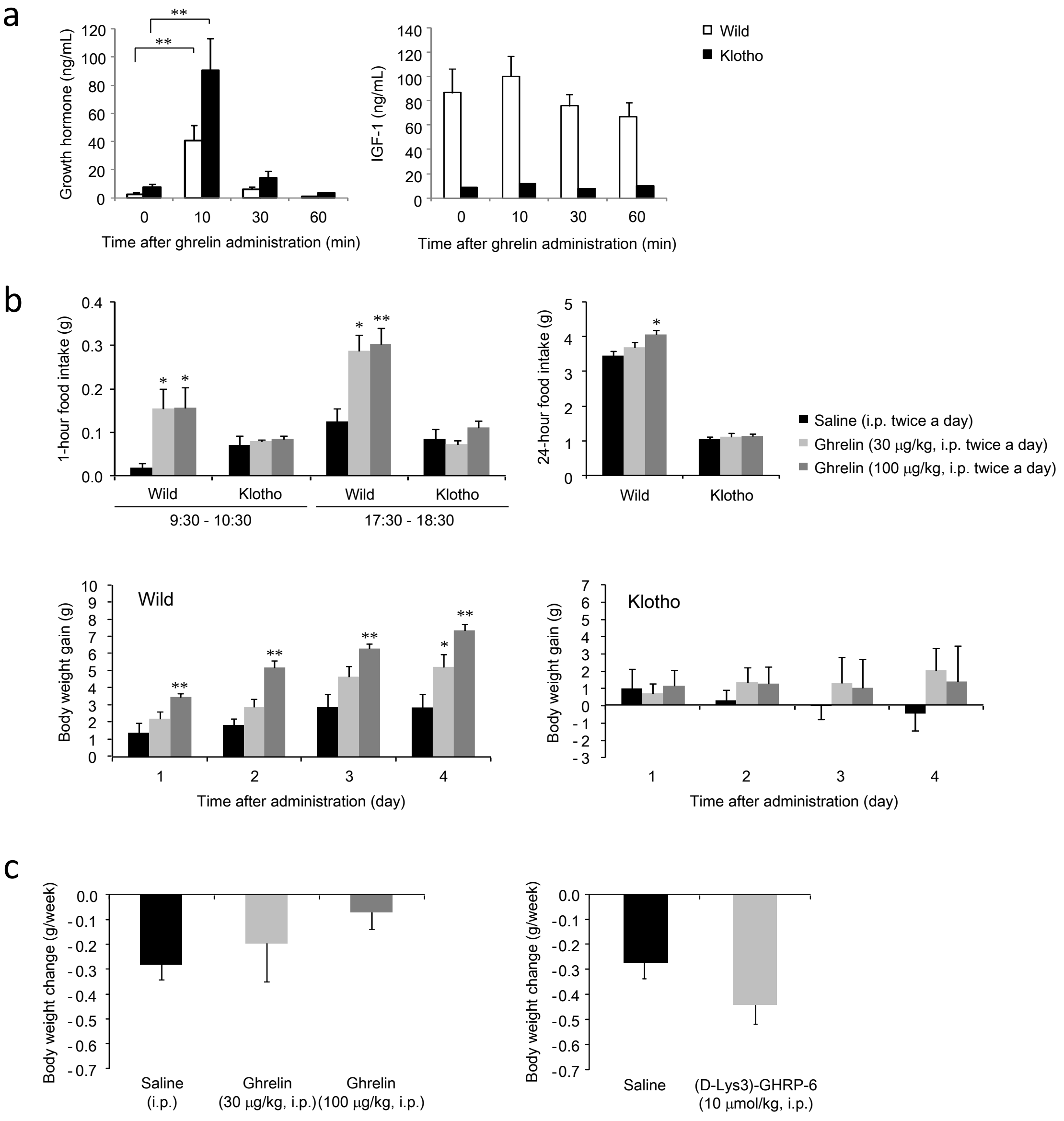

Supplementary Figure 2. Ghrelin resistance in klotho-deficient mice. (**a**) The plasma growth hormone (GH) concentration increased immediately after ghrelin administration (100 µg/kg, i.p.) in wild-type and klotho-deficient mice. No significant effect of ghrelin on the levels of insulin-like growth factor (IGF-1) was observed in either mouse model. ** $P < 0.01$ (n=6-8). (**b**) Ghrelin-induced increases in food intake and body weight were not observed in klotho-deficient mice compared with wild-type mice. * $P < 0.05$, ** $P < 0.01$ (n=8-10). (**c**) No significant effect of ghrelin or ghrelin receptor antagonist (D-Lys3)-GHRP-6 was observed on body weight change in klotho-deficient mice during the survival study.

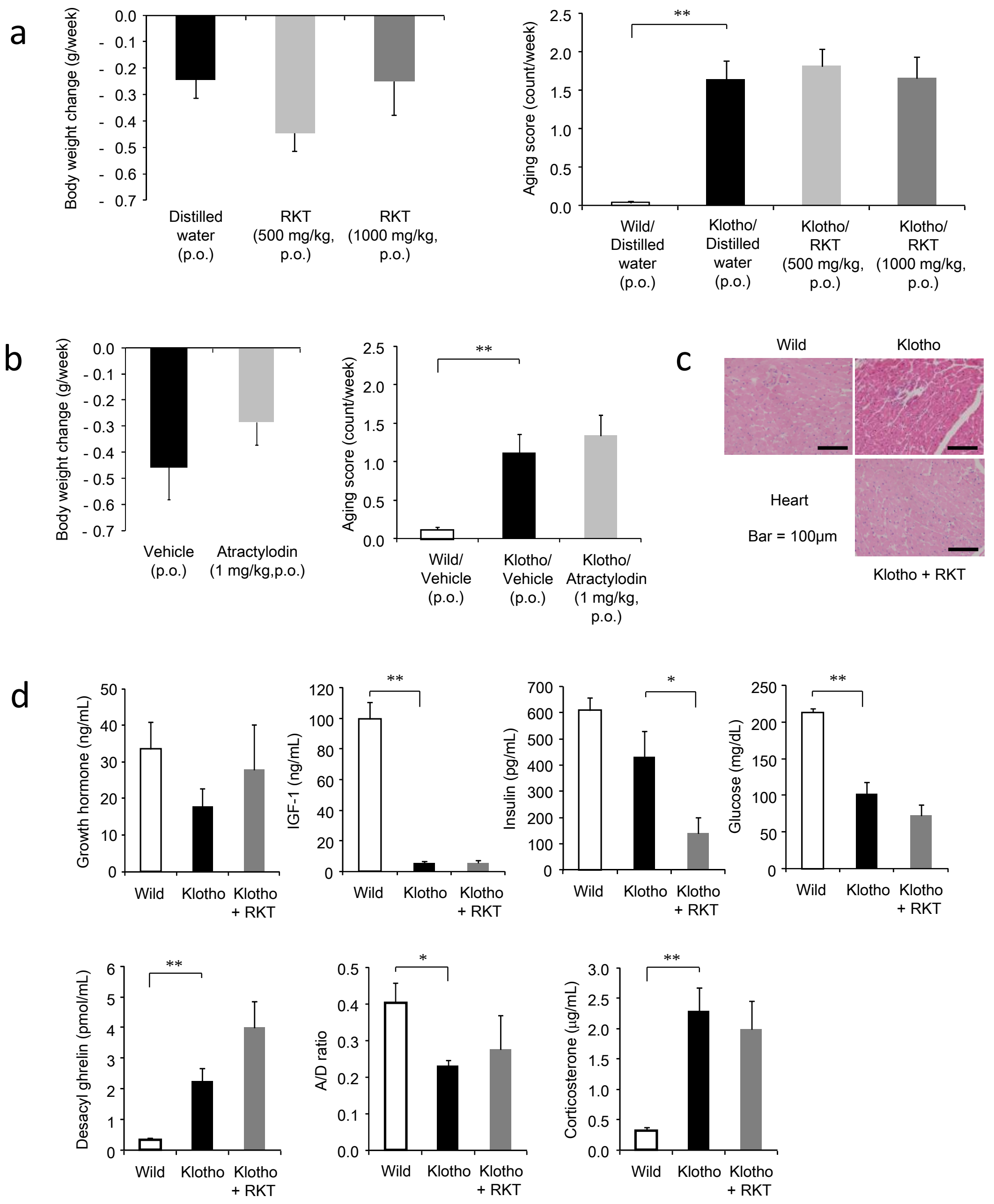

Supplementary Figure 3. Effect of rikkunshito (RKT) and atractylodin on aging in klotho-deficient mice. (**a** and **b**) No significant effect of RKT (500 or 1000 mg/kg, p.o., n=18-20) or atractylodin (1 mg/kg, p.o., n=8-10) on body weight change or aging score in klotho-deficient mice during the survival study was observed. ** P < 0.01. (**c**) Focal atrophy of myocardial fiber of Klotho-deficient mice at the end of the survival study was not observed. (**d**) The plasma desacyl ghrelin and corticosterone concentrations increased, and insulin-like growth factor (IGF-1), glucose, and acyl ghrelin/desacyl ghrelin (A/D) ratio decreased in klotho-deficient mice compared with wild-type. The fed condition is used here because fasting is a severe stress leading to death in this model. There was no significant effect of rikkunshito (RKT; 1000 mg/kg, p.o. for 4 days) treatment on these parameters, except for decreased insulin in the fed condition. * P < 0.05, ** P < 0.01 (n=8-10).

a

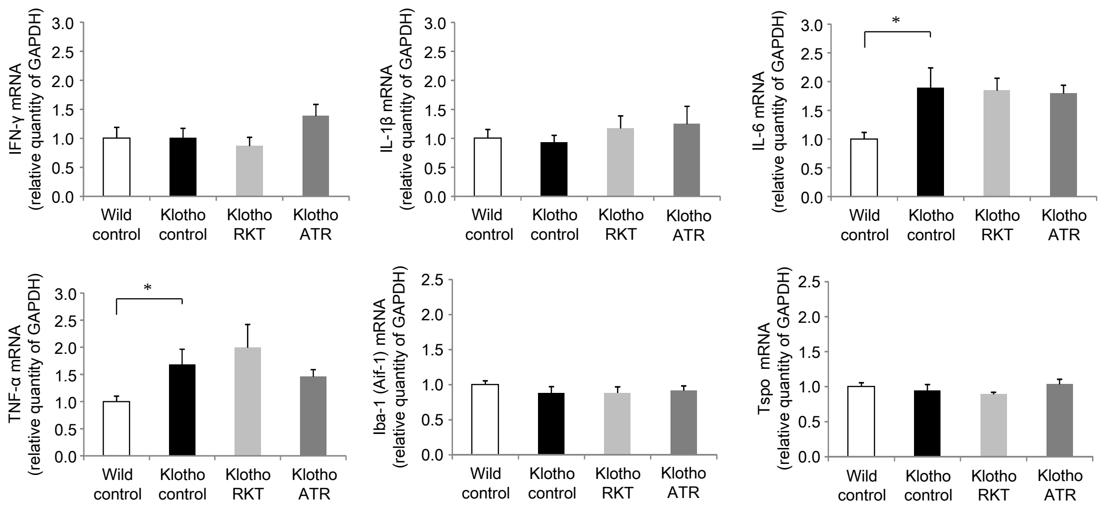

b

Gene symbol	Wild Vehicle	Klotho Vehicle	Klotho RKT	Klotho ATR	Biological function
Gcm1	1.0	0.4	1.2	1.0	DNA demethylation
Peg3	1.0	0.6	1.0	1.4	Inhibition of apoptosis
Ttr	1.0	2.3	0.8	1.4	Induced amyloidosis
Clec4a2	1.0	1.6	0.5	0.4	Negative signals into dendritic cells expansion
srGAP2	1.0	1.4	0.5	0.4	Negatively regulates neuronal migration
Synergin	1.0	1.2	0.5	0.4	Induced apoptosis
Lst1	1.0	1.1	0.4	0.4	Inflammation
Trio	1.0	1.1	0.3	0.2	Neuronal cell death

Ratio

x 3
x 2
x 1.5
x 1/1.5
x 1/2
x 1/3

c

Gene symbol	Vehicle	RKT	ATR
Adcyap1			
Adcyap1r1			
Agrp			
Apln			
Avp			
Avpi1			
Calca			
Cartpt			
Cbln1			
Cbln2			
Cck			
Chga			
Chgb			
Cort			
Creb1			
Creb3			
Crh			
Crhr1			
Crhr2			
Dbi			
Gal			
Grp			
Hcrt			
Hcrtr1			
Htr2c			
Ifngr1			
Igf1			
Igf2r			
Igfbp2			

Gene symbol	Vehicle	RKT	ATR
Nmb			
Npb			
Npy			
Npy1r			
Npy2r			
Nts			
Nucb1			
Nucb2			
Nxph3			
Oxt			
Oxtr			
Pdyn			
Penk			
Pmch			
Pnoc			
Pomc			
Ramp1			
Ramp2			
Rln1			
Scg2			
Scg3			
Scg5			
Sst			
Tac1			
Tac2			
Trh			
Trhr			
Vgf			

Supplementary Figure 4. Hypothalamic inflammatory and appetite-related peptide gene expression in klotho-deficient mice. (**a**) Klotho-deficient mice showed increased hypothalamic gene expression of interleukin-6 (IL-6) and tumor necrosis factor-α (TNF-α), which were not affected by rikkunshito (RKT; 1000 mg/kg, p.o.) and atractylodin (ATR; 1 mg/kg, p.o.) administration for 11 days. There were no differences in gene expression of microglia marker, ionized calcium binding adaptor molecule 1 (Iba-1) and peripheral-type benzodiazepine receptor (Tspo) in hypothalamus. * $P < 0.05$ (n=9-10). (**b** and **c**) Hypothalamic appetite-related peptide gene expression levels were measured using a microarray analysis. Data are shown as the ratio of the expression levels in klotho-deficient mice to wild-type mice. Modest increases in arginine vasopressin (AVP) and POMC and a decrease in IGF-1 after treatment with rikkunshito and atractylodin were observed.

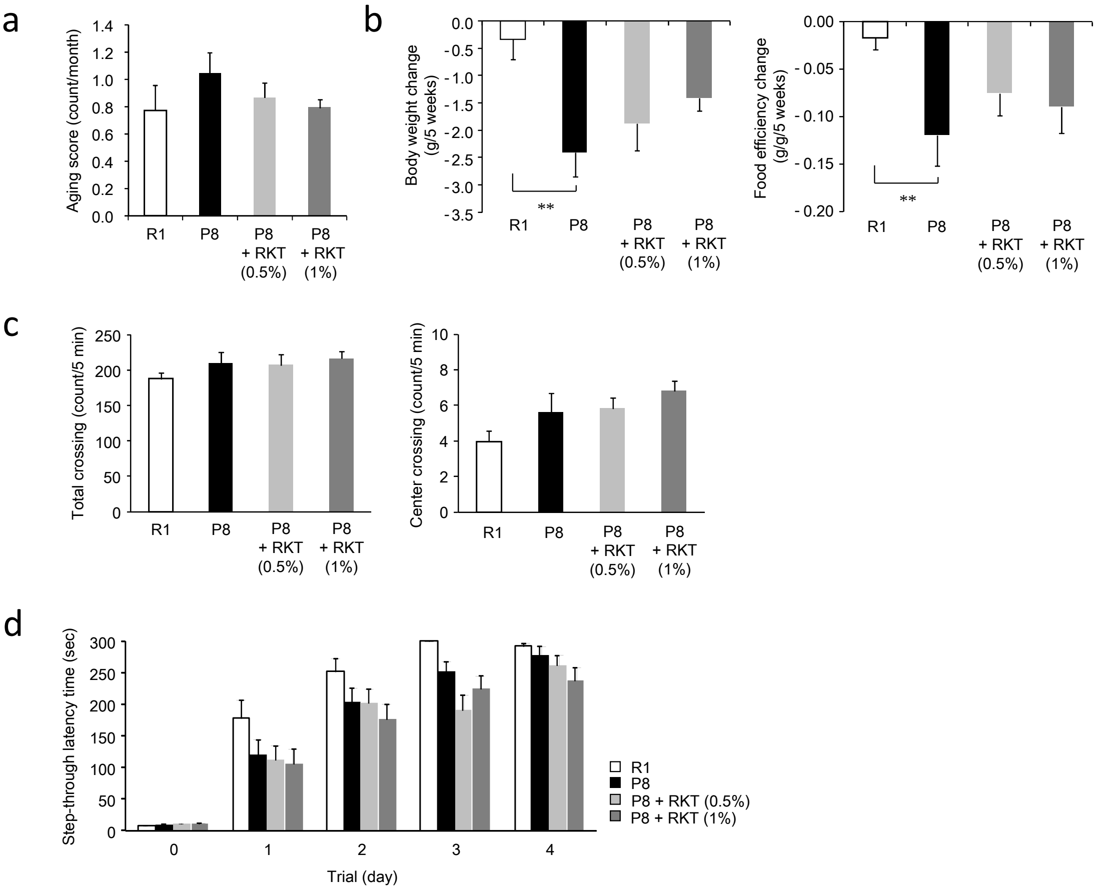

Supplementary Figure 5. Effect of rikkunshito on aging in SAMP8 mice. (**a**) No change in the aging score was observed. (**b**) Twenty-three-week-old SAMP8 (P8) and SAMR1 (R1) mice were given rikkunshito (RKT)-containing chow or control chow. The rates of change in body weight and food efficiency, which were calculated as body weight gain per food intake every five weeks, decreased in SAMP8 mice. These were not affected by RKT treatment. (**c** and **d**) There were no differences in anxiety-like behavior in an open-field test (c) or memory disturbance in a step-through passive-avoidance test (d), although the difference between 39- or 40-week-old SAMP8 and SAMR1 mice failed to reach statistical significance. ** P < 0.01 (n=17-20).

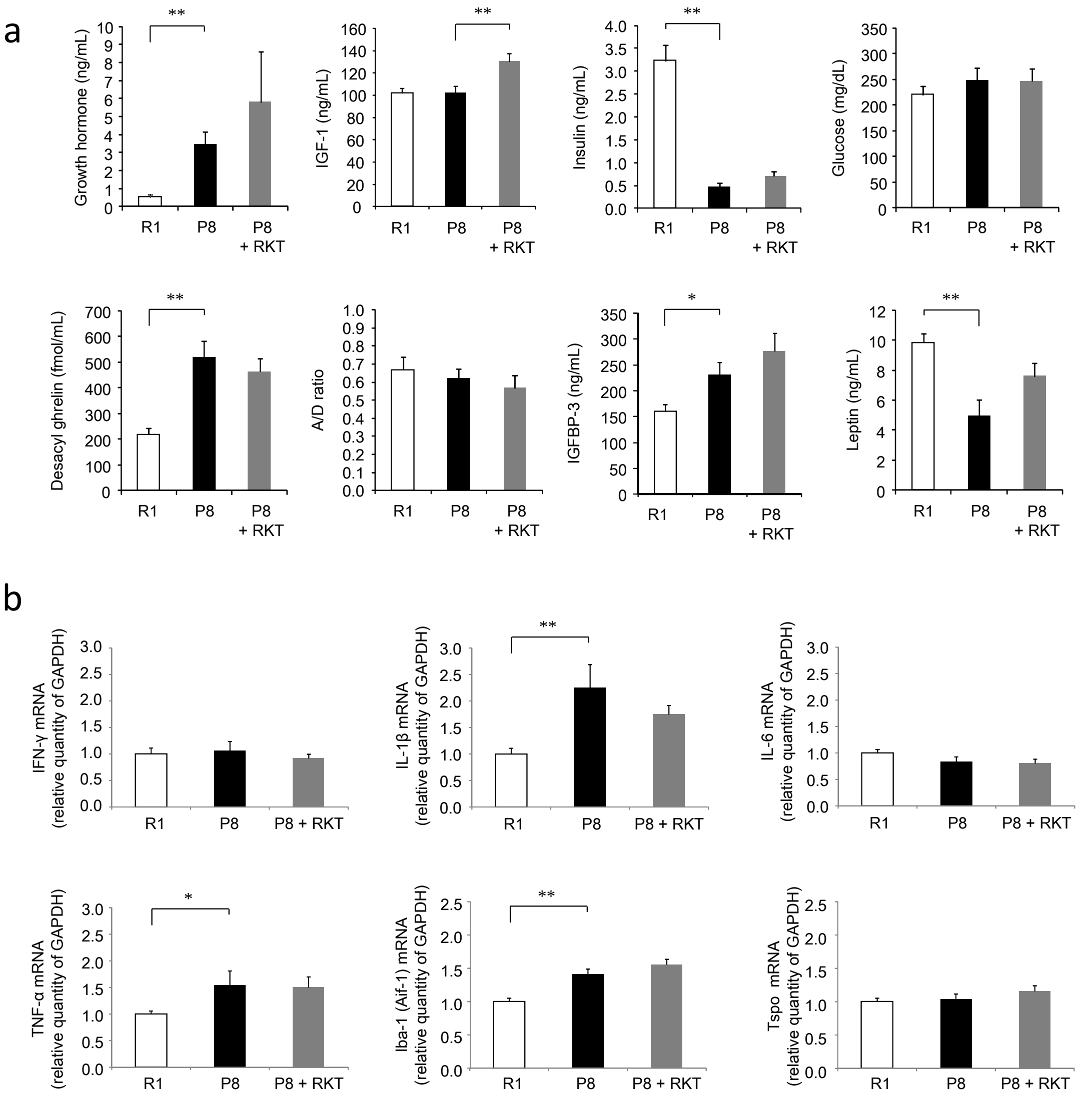

Supplementary Figure 6. Ghrelin-related and inflammatory factors in SAMP8 mice. (**a**) Twenty-three-week-old SAMP8 (P8) and SAMR1 (R1) mice were given rikkunshito (RKT; 1%)-containing chow or control chow for 19 weeks. The plasma concentrations of growth hormone (GH), desacyl ghrelin, and insulin-like growth factor-binding protein (IGFBP-3) increased, and insulin and leptin decreased in P8 mice compared to R1 mice. RKT treatment increased plasma insulin-like growth factor (IGF-1) concentration, but there were no differences between P8 and RKT-treated SAMP8 mice (P8 + RKT) on the other parameters. A/D: acyl ghrelin/desacyl ghrelin. (**b**) SAMP8 mice showed increased hypothalamic gene expression of interleukin-1β (IL-1β), tumor necrosis factor-α (TNF-α), and ionized calcium binding adaptor molecule 1 (Iba-1), which were not affected by RKT (1%) administration. * $P < 0.05$, ** $P < 0.01$ (n=15-19).

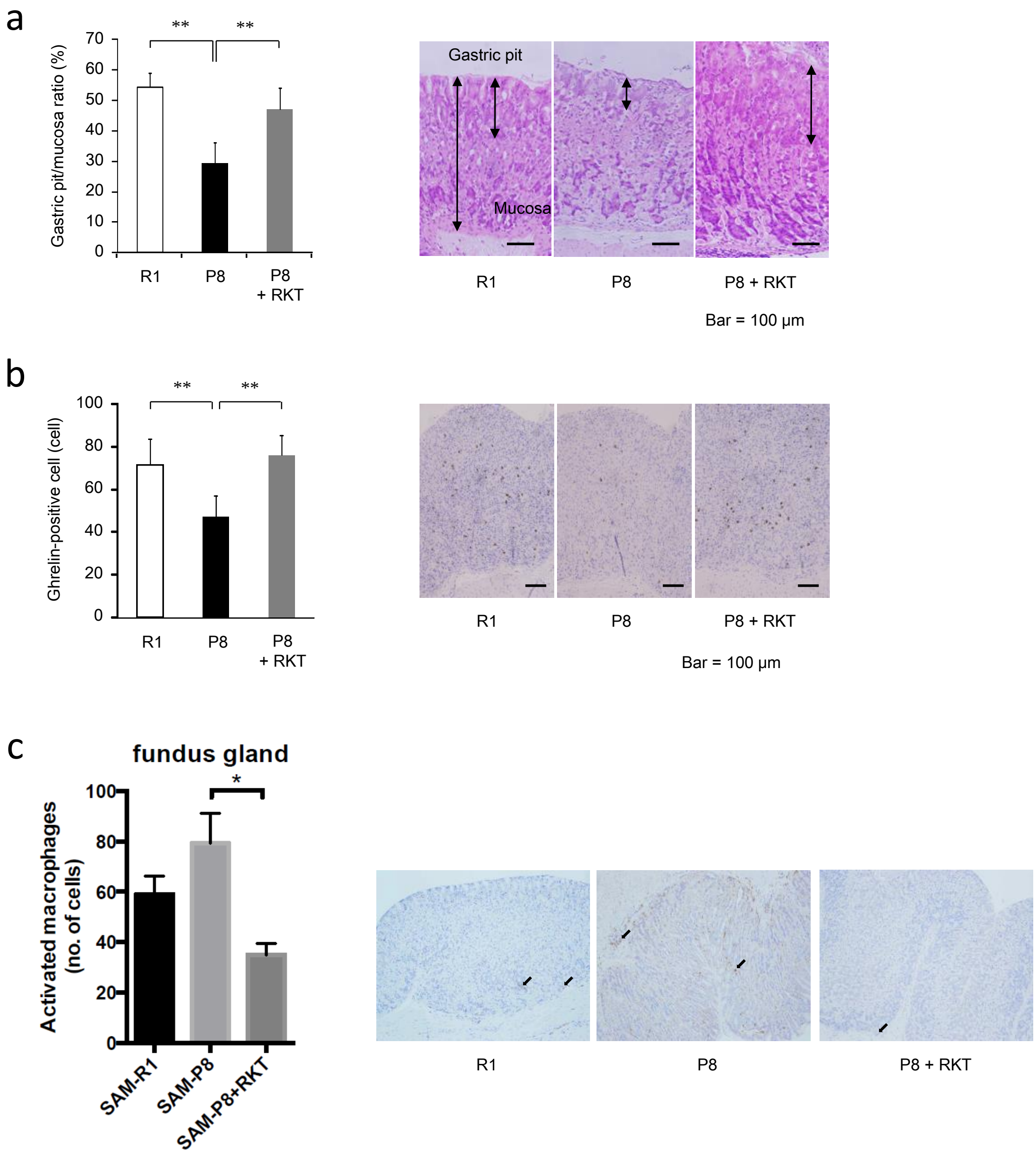

Supplementary Figure 7. Histopathological changes of gastric mucosa in SAMP8 mice. (**a-c**) Twenty-three-week-old SAMP8 (P8) and SAMR1 (R1) mice were given rikkunshito (RKT; 1%)-containing chow or control chow for 19 weeks. Treatment with RKT (1%) inhibited gastric mucosal atrophy (a) and the decrease in the numbers of ghrelin-positive cells (b) in SAMP8 mice (n=15-19). Activated macrophages (Iba-1 positive cells) in fundus gland of SAMP8 mice (c) were decreased by RKT (1%) administration (n=13-15). * $P < 0.05$, ** $P < 0.01$.

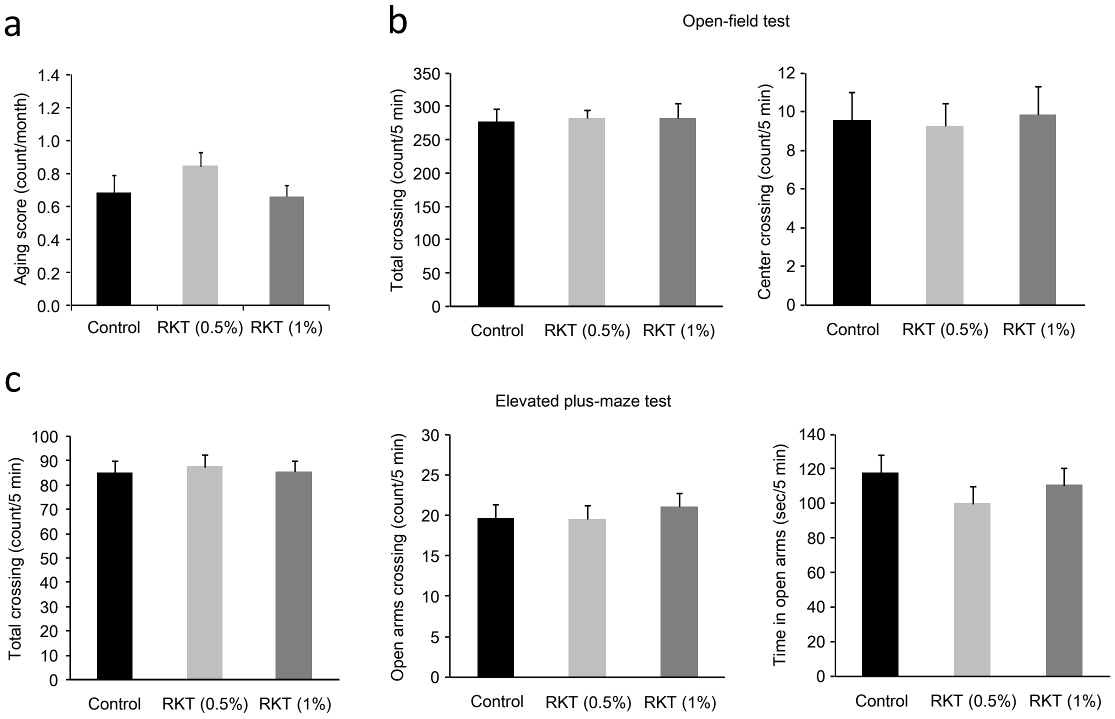

Supplementary Figure 8. Effect of rikkunshito on aging in ICR mice. ICR mice were given rikkunshito (RKT; 0.5% or 1%)-containing chow or control chow. (**a**) No significant effect of RKT on the aging score in ICR mice was observed. (**b** and **c**) Behavioral analyses to evaluate anxiety in ICR mice. Two-month treatment with RKT displayed no anxiolytic action, which was estimated with the open-field test (b) and the elevated plus-maze test (c) (n=18-23).

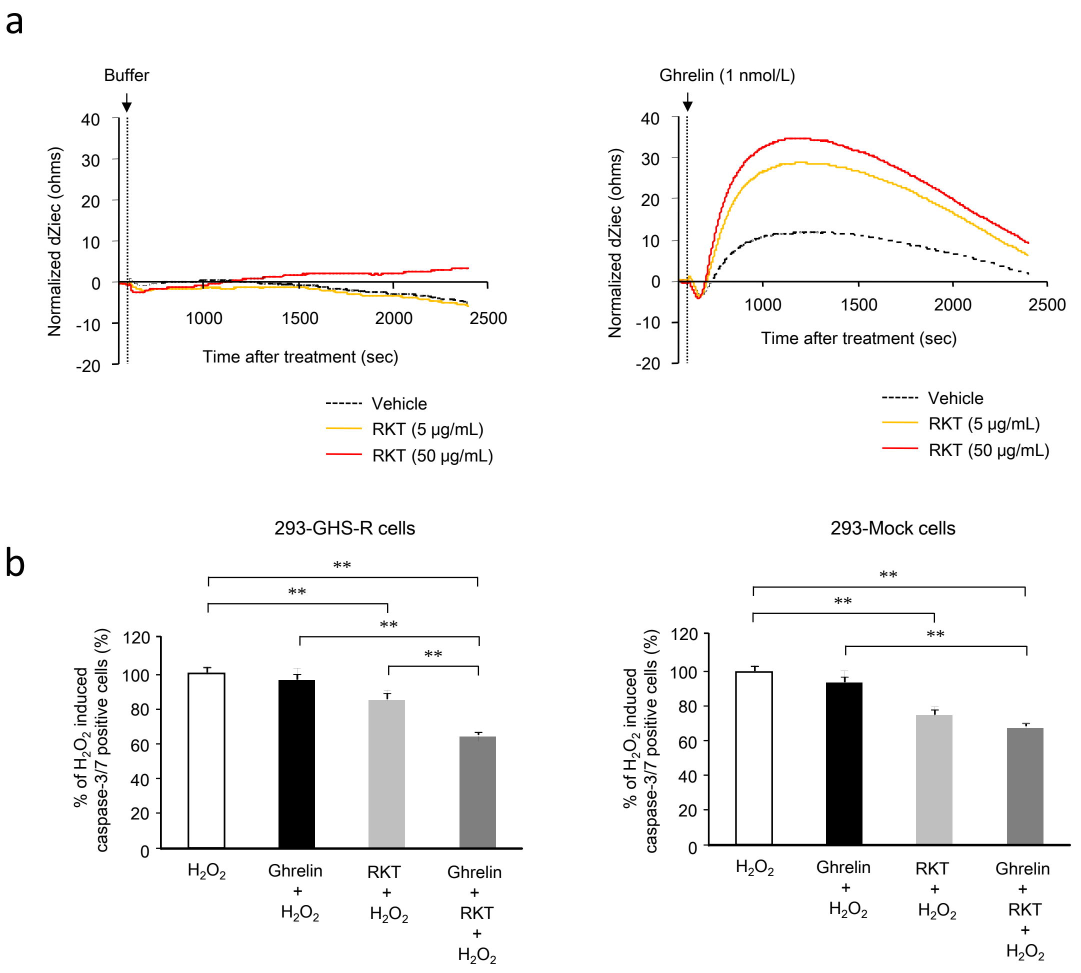

Supplementary Figure 9. Potentiation of the ghrelin receptor signal by rikkunshito (RKT) in GHS-R1a-expressing cells. (**a**) An impedance-based cell assay was conducted using the CellKey^{TM} assay system. HEK293A cells stably expressing human GHS-1a receptor were pretreated with RKT (5 and 50 µg/mL) or vehicle. Then, ghrelin (1 nmol/L) was applied to cells for 600 s, and electrical impedance, which is induced by a change of intracellular signaling, was detected. (**b**) GHS-R1a-expressing HEK293A (293-GHS-R) cells and mock (293-Mock) cells were exposed to H_2O_2 (0.15 mmol/L) and treated with 100 nmol/L ghrelin and/or 100 µg/mL RKT for 20 hours. Cell apoptosis was determined with a caspase-3/7 activity assay. Rikkunshito potentiated the cellular response to ghrelin (a) and inhibited oxidative cell death (b). ** $P < 0.01$ (n=3).

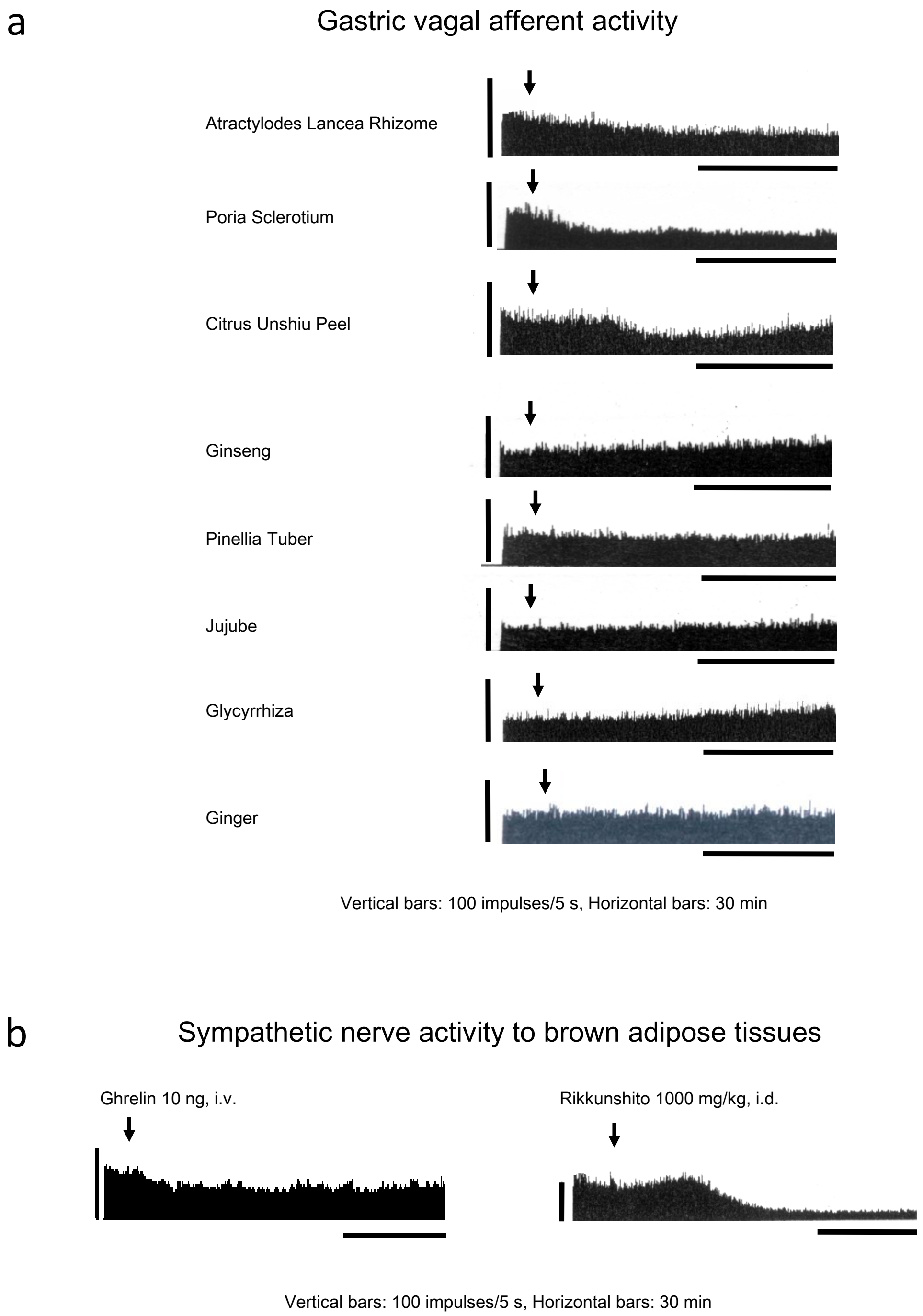

Supplementary Figure 10. Autonomic nervous activity. (**a**) The electrophysiologic study demonstrated that the afferent activities of the gastric vagus nerve were decreased with administration of 400 mg/kg rikkunshito's constituents (atractylodes lancea rhizome, poria sclerotium, and citrus unshiu peel) in urethane anesthetized rats. (**b**) Ghrelin (10 ng) and rikkunshito (1000 mg/kg) decreased the sympathetic nerve activity to brown adipose tissues in urethane anesthetized rats.

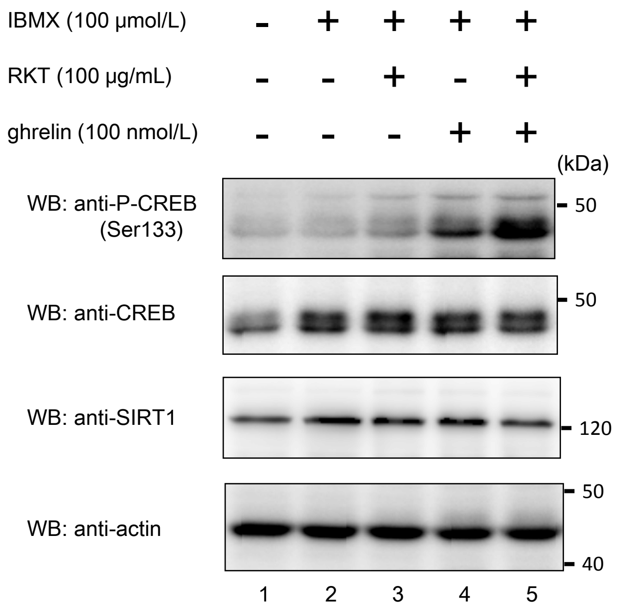

Supplementary Figure 11. Western blotting. 293-GHS-R cells were pre-treated with 100 µmol/L IBMX for 30 min, followed by ghrelin for 30 min, and rikkunshito for 90 min. Ghrelin (100 nmol/L) increased phosphorylated CREB (P-CREB) in 293-GHS-R cells and it was augmented by rikkunshito (RKT; 100 µg/mL).

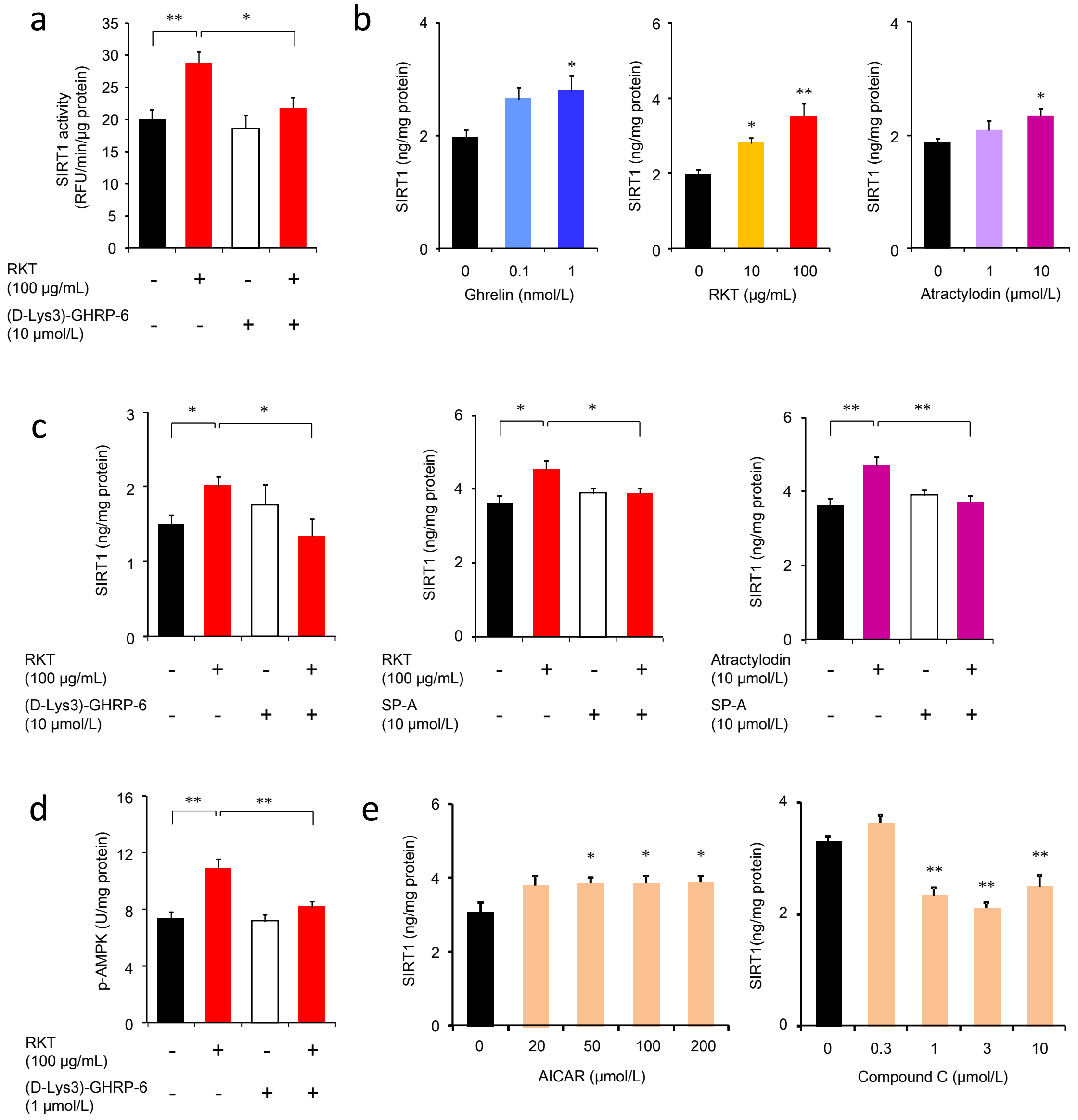

Supplementary Figure 12. The effects of ghrelin signaling on sirtuin1 (SIRT1) and phosphorylated adenosine monophosphate-activated protein kinase (AMPK) expression in human umbilical vein endothelial cells (HUVECs). (**a**) Rikkunshito (RKT) upregulated SIRT1 activity in HUVECs, which was blocked by the ghrelin antagonist (D-Lys3)-GHRP-6. * P < 0.05, ** P < 0.01 (n=6). (**b** and **c**) Ghrelin, RKT, and atractylodin elicited increases in SIRT1 protein expression in HUVECs. The effect of RKT and atractylodin was inhibited by treatment with (D-Lys3)-GHRP-6 or GHS-R inverse agonist (SP-A). * P < 0.05, ** P < 0.01 (n=6). (**d**) RKT upregulated phosphorylated AMPK expression in HUVECs, which was blocked by (D-Lys3)-GHRP-6. ** P < 0.01 (n=6). (**e**) The SIRT1 protein expressions in HUVECs were increased by AMPK activator AICAR and decreased by AMPK inhibitor Compound C. * P < 0.05, ** P < 0.01 (n=6). The levels of SIRT1 protein and phosphorylated AMPK in cell lysate were measured using an enzyme-linked immunosorbent assay in this study.

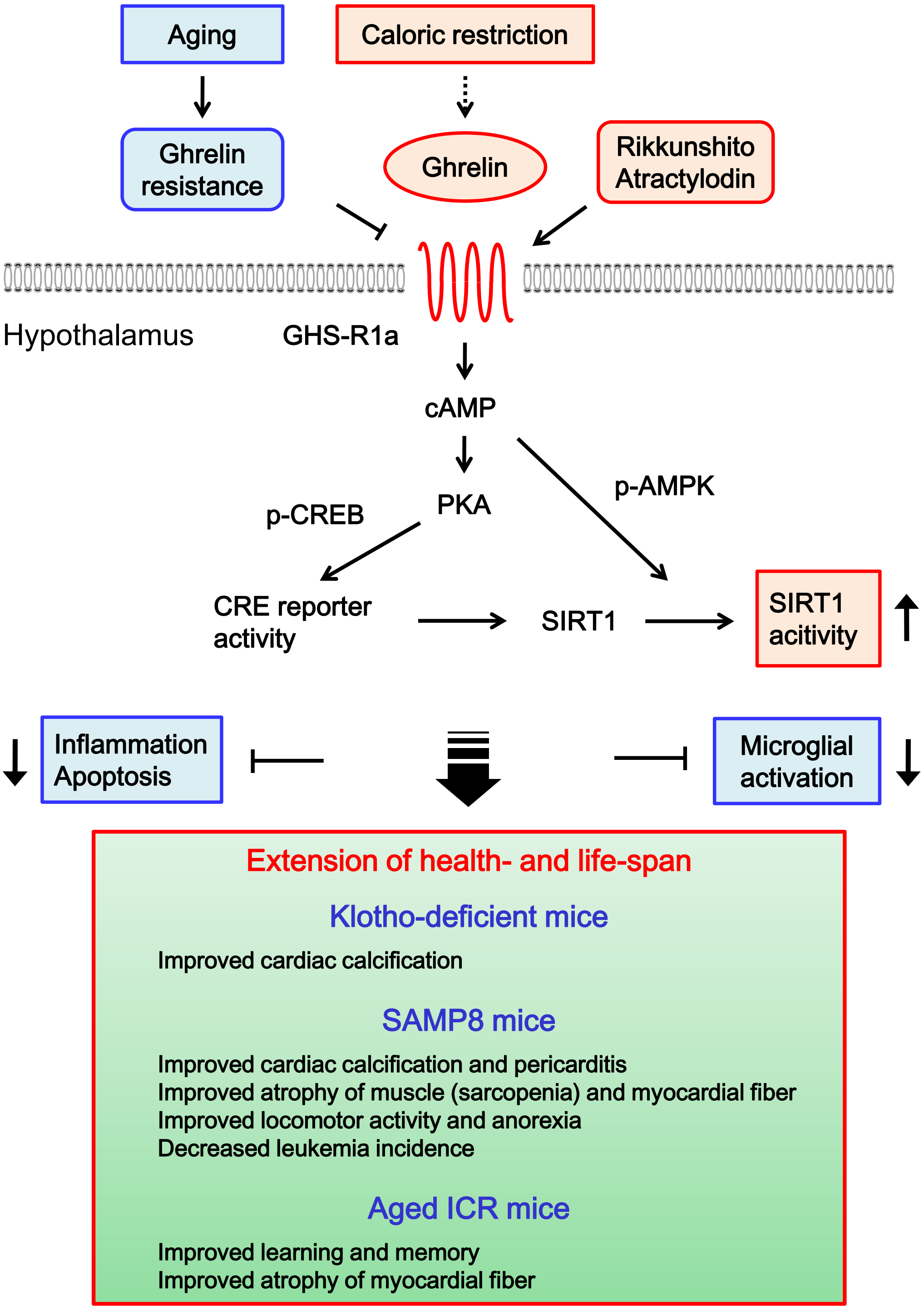

Supplementary Figure 13. Ghrelin signaling as a mimetic of caloric restriction (CR). Ghrelin and the ghrelin signaling potentiators rikkunshito and atractylodin increased sirtuin1 (SIRT1) activity through cAMP-CREB pathway or phosphorylated adenosine monophosphate-activated protein kinase (AMPK) in GHS-R expressing cells. Rikkunshito increased hypothalamic SIRT1 activity and ameliorated inflammatory activation of microglia, leading to the improvement on age-related diseases and survival in klotho-deficient mice, SAMP8 mice, and ICR mice, three different animal models on human aging that are characterized by ghrelin resistance. These results indicate that ghrelin secreted from stomach in response to fasting and CR may underlie the beneficial effects of CR on aging through SIRT1 pathways in the hypothalamus. The potentiation of ghrelin signaling may be useful to delay age-related diseases and functional decline, and to extend health- and life-span.

Supplementary Table 1. Causes of death in klotho-deficient mice.

% animals	Wild	Klotho	Klotho + RKT (1000 mg/kg, p.o.)
	n=14	n=14	n=14
Calcification			
Heart	0%	79% **	43%
Stomach	0%	100% **	100%
Aorta	0%	100% **	100%
Kidney	0%	100% **	100%
Others			
Spleen			
Atrophy	0%	86% **	86%
Thymus			
Involution	0%	79% **	71%
Mesenteric lymph node			
Atrophy	0%	43% **	7%

The pathology of mice that died or were euthanized at the end of the experimental period in a survival study was observed. Calcification was observed in several tissues, such as the heart, stomach, aorta, and kidney of klotho-deficient mice. Histological score of calcification in heart is shown in Figure 1f. These findings suggest that a major cause of death in klotho-deficient mice was systemic calcification. Other pathological changes in the spleen, thymus, and mesenteric lymph node of klotho-deficient mice were also observed. ** $P < 0.01$ vs. Wild.

Supplementary Table 2. Causes of death in SAMR1 and P8 mice.

% animals	R1<br>n=17	P8<br>n=20	P8 + RKT (1%)<br>n=20
Leukemia	71%	80%	40% ##
Others			
Tumor	12%	5%	20%
Myocardial infarction	6%	0%	0%
Others	12%	15%	40%

The observation of pathology after death in the survival study showed that the main cause of death was leukemia in SAMR1 (R1) and SAMP8 (P8) mice. Rikkunshito (RKT; 1%) treatment decreased the occurrence of leukemia in SAMP8 mice. ## $P < 0.01$ vs. P8.

Supplementary Table 3. Causes of death in ICR mice.

% animals	ICR<br>n=22	ICR + RKT (1%)<br>n=23
Tumor	64%	74%
Lung	32%	52%
Liver	32%	22%
Others		
Myocardial infarction	5%	0%
Hepatic cirrhosis	0%	4%
Others	32%	22%

The observation of pathology after death in the survival study showed the development of tumors in the lung and liver of ICR mice. There was no significant difference in the occurrence of tumors between ICR and rikkunshito (RKT; 1%)-treated ICR mice.
